# TARG1 protects against toxic DNA ADP-ribosylation

**DOI:** 10.1093/nar/gkab771

**Published:** 2021-09-11

**Authors:** Callum Tromans-Coia, Andrea Sanchi, Giuliana K Moeller, Gyula Timinszky, Massimo Lopes, Ivan Ahel

**Affiliations:** Sir William Dunn School of Pathology, University of Oxford, Oxford OX1 3RE, UK; Institute of Molecular Cancer Research, University of Zurich, 8057 Zurich, Switzerland; Department of Physiological Chemistry, Biomedical Center (BMC), Faculty of Medicine, LMU Munich, 82152 Planegg-Martinsried, Germany; Lendület Laboratory of DNA Damage and Nuclear Dynamics, Institute of Genetics, Biological Research Centre, Eötvös Loránd Research Network (ELKH), 6276 Szeged, Hungary; Institute of Molecular Cancer Research, University of Zurich, 8057 Zurich, Switzerland; Sir William Dunn School of Pathology, University of Oxford, Oxford OX1 3RE, UK

## Abstract

ADP-ribosylation is a modification that targets a variety of macromolecules and regulates a diverse array of important cellular processes. ADP-ribosylation is catalysed by ADP-ribosyltransferases and reversed by ADP-ribosylhydrolases. Recently, an ADP-ribosyltransferase toxin termed ‘DarT’ from bacteria, which is distantly related to human PARPs, was shown to modify thymidine in single-stranded DNA in a sequence specific manner. The antitoxin of DarT is the macrodomain containing ADP-ribosylhydrolase DarG, which shares striking structural homology with the human ADP-ribosylhydrolase TARG1. Here, we show that TARG1, like DarG, can reverse thymidine-linked DNA ADP-ribosylation. We find that TARG1-deficient human cells are extremely sensitive to DNA ADP-ribosylation. Furthermore, we also demonstrate the first detection of reversible ADP-ribosylation on genomic DNA *in vivo* from human cells. Collectively, our results elucidate the impact of DNA ADP-ribosylation in human cells and provides a molecular toolkit for future studies into this largely unknown facet of ADP-ribosylation.

## INTRODUCTION

The DNA damage response (DDR) is a critical and intricate signaling pathway that harmonises cellular events in order to repair damaged DNA. The intricacy of the DDR is in part a result of the variety of DNA damage types, from both endogenous and exogenous sources, that it must repair. DNA damage can arise as alterations in the chemical structure of DNA, such as base adducts or as breaks in either one or both of the DNA strands. In any case, the DDR must first detect the specific type of damage and then regulate the various ways in which the damage can be repaired. This is performed in tandem with the regulation of cell metabolism, such as translation suppression or cell cycle arrest, with the ultimate goal of maintaining genome integrity.

The bases of DNA can be damaged in numerous ways, such as alkylation and oxidation, and can be repaired by the base excision repair pathway (BER). This pathways involves the recognition of base aberrations by damage-specific glycosylases that remove the damaged base, followed by cleavage of the DNA backbone. DNA polymerases then replace the missing DNA and DNA ligases seal the DNA backbone to restore the original DNA sequence (1). Bulkier DNA lesions that distort the DNA helix, such as large hydrocarbon adducts from tobacco smoke, can be repaired by the nucleotide excision repair pathway (NER) (2). This pathway involves the excision of an oligonucleotide containing the aberrant base. The resulting gap in the DNA is filled by DNA polymerases and sealed by DNA ligases in order to complete the repair (3). A discontinuity in one strand of DNA is known as a single-strand break (SSB) and can arise as a result of reactive oxygen species. SSBs can also be generated during the aforementioned BER following DNA alkylation (4). SSBs can be recognised by poly(ADP-ribose) polymerase 1 (PARP1) or PARP2 that upon DNA binding are activated and catalyse the formation of ADP-ribosylation, introduced later (5–7). The ADP-ribosylation modification then recruits important SSB repair factors (8–11). Breaks in both strands of DNA in close proximity are defined as double-strand breaks (DSBs) and can be repaired by a variety of pathways depending on the cellular context, such as cell cycle phase. The predominant repair pathways are non-homologous end joining and homologous recombination, both of which ensure that potentially clastogenic DSBs are effectively repaired (12,13).

In addition to these complex, multi-stage DNA repair pathways, requiring the temporal and spatial recruitment of multiple proteins, the DDR also possesses single repair enzymes that can directly repair certain forms of DNA damage by direct reversal. These repair factors offer a comparatively simple and effective way of repairing DNA lesions without requiring DNA synthesis, incision into the DNA backbone or a nucleotide template, unlike the aforementioned pathways (14,15). Notably, certain types of base alkylations can be directly reversed by alkyltransferases or dioxygenases (4,14,16). UV adducts can also be repaired directly by lesion specific photolyases, although human cells do not possess a photolyase homolog and instead rely on the aforementioned NER pathway (14,17,18). These simple, error-free direct repair enzymes help to ensure the efficient preservation of genome integrity.

Sophisticated regulation of the more complex DDR pathways is in part enabled by ADP-ribosylation, a dynamic chemical modification of macromolecules that can be found across all domains of life (19,20). This modification involves the enzymatic transfer of an ADP-ribose moiety from NAD^+^ onto target substrates with the simultaneous release of nicotinamide. ADP-ribosylation on target substrates can be in either mono or poly-ADP-ribose forms. The enzymes that catalyse these reactions are collectively known as ADP-ribosyltransferases (ARTs), with the aforementioned PARP1 being the founding and most intensely studied member of this class of enzymes (7,21). The dynamic nature of ADP-ribosylation is enabled by the action of a range of ADP-ribosylhydrolases that can remove either the mono, poly or both forms of the modification. The principal ADP-ribosylhydrolase in human cells is poly(ADP-ribosyl) glycohydrolase (PARG), which can rapidly remove poly-ADP-ribosylation (22,23). However, PARG is unable to cleave the linkage between target proteins and the proximal ADP-ribose unit. Instead, the final ADP-ribose can be removed by macrodomain containing ADP-ribosylhydrolases such as MacroD1, MacroD2 or TARG1 (24). Under conditions of DNA damage, the catalytic activity of PARP1 or PARP2 can be targeted towards specific serine residues in target proteins when in complex with the cofactor HPF1 (25–27). The ADP-ribosylation of serine can only be reversed by the ADP-ribosylhydrolase ARH3 (28). The combined action of both ARTs and ADP-ribosylhydrolases enables ADP-ribosylation to serve as a dynamic signaling mechanism that can control the activity of a range of proteins involved within the DDR, in addition to regulating chromatin organisation, transcription and cell division.

The ADP-ribosylhydrolases PARG, MacroD1, MacroD2 and TARG1 contain macrodomains that are the source of their catalytic activity. Interestingly, the macrodomain of TARG1 has been suggested to have a catalytic mechanism that is distinct from the hydrolases PARG and MacroD1 (29). The catalytic dyad of TARG1 has been compared to the core catalytic residues of the DNA glycosylase OGG1 (30). *In vitro*, TARG1 has been shown to cleave mono and poly-ADP-ribosylation from glutamate and aspartate residues and patients that are deficient in TARG1 have been shown to develop a neurodegenerative disorder (30). However, despite a link with neurodegeneration, the physiological role of TARG1 remains enigmatic. Recent studies have shown that overexpressed TARG1 shuttles between nucleoli and the nucleoplasm and re-locates to sites of DNA damage in a PAR-dependent manner (30,31). Additionally, TARG1 has been suggested to interact with ribosomes and ribosome biogenesis factors but the role of these interactions in unknown (31). Loss of TARG1 has also been associated with elevated levels of transcription, but the pathophysiological relevance of this remains to be determined (32).

Traditionally, ADP-ribosylation has been studied as a protein modification. However, growing research is beginning to show that nucleic acids can also be ADP-ribosylated (33). The first reported DNA modifying ART was pierisin from the cabbage butterfly (34). Pierisin and its orthologues from molluscs and bacteria were shown to mono-ADP-ribosylate guanine bases in double-stranded DNA (dsDNA) and were proposed to be involved in parasite defence and metamorphosis. Introducing pierisin into HeLa cells led to DNA damage and apoptosis (35,36). Eukaryotic PARPs, which are known mostly for performing protein modification, have also been shown to ADP-ribosylate nucleic acids. Recent work has revealed that, *in vitro*, PARP1-3 can ADP-ribosylate the phosphate groups found at DNA ends (37–40). It has been suggested that in the correct DNA context, PARP1 may have a preference for the ADP-ribosylation of 3′-phosphates over its own auto-modification, suggesting that this could potentially be an abundant modification (41). It has also been revealed that the ADP-ribosylhydrolases PARG, ARH3, MacroD2 and TARG1 are able to reverse PARP1 or PARP3-mediated phosphate-linked DNA ADP-ribosylation *in vitro (**38**)*. TRPT1/KptA, an ART closely related to the PARP enzymes, was also shown to reversibly ADP-ribosylate 5′-phosphates on DNA and RNA ends (42). Similar activity is observed for TRPT1 homologues in lower organisms (42,43). Furthermore, PARP10, PARP11 and PARP15 have been shown to ADP-ribosylate the 5′-phosphate at RNA ends (42). However, the role of nucleic acid ADP-ribosylation and the impact of this modification in cells is unclear.

Recently, a novel DNA modifying PARP-like bacterial toxin termed DarT (‘DNA ADP-ribosyltransferase’) was reported (44). DarT can be found in thermophiles such as *Thermus aquaticus* and global pathogens such as *Mycobacterium tuberculosis* and enteropathogenic *Escherichia coli* (44–46). DarT catalyses the transfer of a single ADP-ribose unit onto thymidine bases on single-stranded DNA (ssDNA) in a sequence-specific manner and shows no activity towards dsDNA, RNA or protein (44–46). DarT is also the toxin in a toxin:antitoxin system, with the antitoxin being DarG, a macrodomain containing ADP-ribosylhydrolase that can reverse thymidine-linked ADP-ribosylation. In *E. coli*, the activity of DarT has been shown to induce the DDR (44–46). In the absence of DarG, thymidine-linked ADP-ribosylation catalysed by an attenuated DarT mutant can be repaired by RecF-mediated homologous recombination in cooperation with NER (45). Depletion of DarG in *Mycobacterium tuberculosis* also results in triggering of the DDR and bacterial cell death (47). The physiological role of DarT in bacteria has been proposed to be a mechanism for inducing dormancy and enabling bacterial persistence through transient blockage of DNA replication in response to challenging environmental pressures or antibiotics (44,48). Indeed, it was recently shown in *M. tuberculosis* that DarT can control bacterial growth by ADP-ribosylating ssDNA at chromosomal replication origins (46).

Interestingly, the molecular structure of the catalytic macrodomain of the antitoxin DarG is strikingly similar to that of the human ADP-ribosylhydrolase TARG1 (30,44). Due to this similarity, we sought to uncover if TARG1 can also reverse thymidine-linked ADP-ribosylation. Here, we show that TARG1 can indeed reverse thymidine-linked ADP-ribosylation of DNA and can also rescue DarT toxicity in bacteria, similar to that of DarG. Due to TARG1 being a human ADP-ribosylhydrolase, we next investigated the impact of DarT-mediated DNA ADP-ribosylation in human cells. To do so, we created a system for heterologous expression of DarT to determine the impact extreme DNA ADP-ribosylation can impose. By using DarT as a genotoxin, we reveal that TARG1-deficient cells are uniquely sensitive to thymidine-linked ADP-ribosylation. This DNA adduct affects replication fork progression and leads to extensive DNA damage signalling in replicating cells that can be rescued by TARG1 overexpression. This reveals the unique and non-redundant catalytic activity of TARG1 in reversing thymidine-linked ADP-ribosylation. Furthermore, this contrasts the removal of ADP-ribose from terminal phosphates on nucleic acids that can be performed by multiple ADP-ribosylhydrolases (38). We also provide evidence for the first detection of reversible ADP-ribosylation on genomic DNA (gDNA) from human cells. Collectively, we demonstrate that thymidine-linked ADP-ribosylation is a novel DNA adduct that leads to replication stress and requires the direct DNA damage repair factor TARG1 for its resolution.

## MATERIALS AND METHODS

### Oligonucleotides

GJ1: GAGCTGTACAAGTCAGATCTCGAGCTC
27mer: CACGACACGAGCAGGCATGTCCACGTG

### Plasmids

Plasmids used here include: pBAD33-V5-DarT (*Thermus aquaticus*), pET28a-6xHis-TARG1-WT, pET28a-6xHis-TARG1-K84A, pET28a-6xHis-DarG-WT-1–155aa (*Thermus aquaticus*), pET28a-6xHis-DarG-K80A-1–155aa (*Thermus aquaticus*), pLIX_403-GFP-DarT-WT (*Thermus aquaticus*), pLIX_403-GFP-DarT-E160A (*Thermus aquaticus*), pLX304-TARG1-WT, pLX304-TARG1-K84A. pLIX_403 and pLX304 were gifts from David Root (pLIX_403 Addgene: #41395, pLX304 Addgene: #25890)

### Thymidine-linked ADP-ribose DNA purification

Unmodified GJ1 or 27mer DNA oligonucleotides at a concentration of 0.5 mM were incubated with 1 μM *Thermus aquaticus* DarT, 5 mM EDTA, ADP-ribosylation buffer (50 mM Tris–Cl pH 8, 150 mM NaCl) and 0.5 mM NAD^+^, made up to a volume of 50 μl with water. Reactions were then incubated overnight at 37°C. DarT ADP-ribosylated DNA oligonucleotides were purified by denaturing urea PAGE and gel pieces containing ADP-ribosylated DNA were extracted using UV shadow. Gel pieces were incubated at 50°C in 500 μl TE buffer for 10 minutes, three times. TE buffer containing ADP-ribosylated DNA was pooled, passed through a 0.2 μM filter and desalted with G-25 spin columns (GE Healthcare). Concentration for ADP-ribosylated DNA was determined by nanodrop using A260 and Beer–Lambert law with a 13 500 μM^–1^cm^–1^ extinction coefficient for ADP-ribose. If required, DNA was further concentrated using a speedvac.

### De-ADP-ribosylation assay

DarT ADP-ribosylated oligonucleotides were used as a substrate at 2 μM. The indicated ADP-ribosylhydrolases were added at a concentration of 1 μM for 30 min at 37°C. Samples were then heated to 95°C for 3 min in 2× TBE urea sample buffer (8 M urea, 1 mM EDTA pH 8, 20 mM Tris pH 8 and 0.05% bromophenol blue). Samples were then loaded onto a prerun denaturing urea PAGE gel containing 8 M urea, 1× TBE and 20% polyacrylamide. The gel was ran at 10–12W in 1× TBE and then stained with ethidium bromide. DNA was visualised by UV using a gel documentation system.

### Bacterial toxicity rescue assay

BL21 DE3 cells were transformed with the indicated plasmids and grown in liquid culture supplemented with 0.8% glucose with the appropriate antibiotics overnight. Cultures were then spread onto LB agar containing the appropriate antibiotics and either 0.8% glucose or 0.8% arabinose with or without 50 μM IPTG. Plates were incubated overnight at 37°C. For western blot analysis of DarT and ADP-ribosylhydrolase expression, cultures were induced with 0.8% arabinose and 50 μM IPTG for 1 h at 37°C. Bacteria were pelleted at 800 × g for 3 min, washed in PBS, pelleted again before being resuspended in boiling lysis buffer (1% SDS, 10 mM Tris, 1 mM EDTA, pH 8) and heated to 95°C for 3 min. Lysates were then prepared for western blot analysis as indicated by the western blot method.

### Cell culture

All cells were grown in a sterile environment and routinely checked for the presence of mycoplasma. U-2 OS or HEK293T (hereafter 293T, used for lentivirus generation only) cells were grown under standard cell culture conditions (humidified atmosphere and 5% CO_2_) in Dulbecco's modified Eagle's medium (DMEM. Sigma, D6429) supplemented with 10% fetal bovine serum (Sigma) and penicillin-streptomycin antibiotics (Thermo Fisher). Unless stated otherwise, the following drugs were used at the indicated final concentrations: hydroxyurea (2 mM, Sigma), camptothecin (1 μM, Selleckchem), MMS (2 mM, Sigma), olaparib (1 μM, Selleckchem), veliparib (1 μM, Selleckchem), doxycycline (concentration varied to match GFP expression between cell lines, Sigma), PARGi (PDD00017273, 1 μM, Torcis). TARG1 knockout cell lines were generated by CRISPR/Cas9 following the published protocol (49). The guide RNA sequences for TARG1 were identified using the gRNA design tools provided by the Zhang lab (http://crispr.mit.edu) (50). The gDNA sequence used was as follows: FWD: caccgAGGATTGTCGCATGGGCGCT; REV: aaacAGCGCCCATGCGACAATCCTc. Annealed primers were cloned into pSpCas9(BB)-2A-GFP (PX458) and the sequence verified plasmid was transfected into U-2 OS cells. 1–2 days post-transfection, GFP-positive single cells were sorted with a FACSAria II into 96-well plates. Monoclonal cell lines were tested for TARG1 deficiency by anti-TARG1 western blot. pSpCas9(BB)-2A-GFP (PX458) was a gift from Feng Zhang (Addgene plasmid #48138).

### Lentiviral transduction

Lentivirus production was carried out by transfecting 293T cells with pCMV-VSV-G (1 μg) and pCMV-dR8.2 dvpr (5 μg) in combination with DarT-pLIX_403 or TARG1-pLX304 (5 μg) using polyfect transfection reagent (QIAgen). 293T cells were transfected for 12 h before the media was replaced and incubated for a further 48 h. This lentivirus containing media was collected and filtered through a 0.45 μM filter and a 2-fold dilution was added to target U-2 OS cells for 48 h. U-2 OS media was then replaced without selection antibiotic and incubated for a further 24 h. Media was then changed for selection antibiotic-containing media (puromycin 0.1 μg/ml for pLIX_403 or blasticidin 1 μg/mL for pLX304) and incubated until the control cells died. Surviving cells from the lowest viral dilution were expanded and monoclones were selected using cloning discs (Sigma). Monoclones were then tested for expression (doxycycline for pLIX_403, no induction necessary for pLX304) and clones with similar levels of expression were taken forward for subsequent experiments.

### Antibodies

Primary antibodies used here were: β-tubulin (ab6046, Abcam), TARG1 (25249–1-AP, Proteintech), γH2AX (05–636, Merck), RPA2 (ab2175, Abcam), RPA2 pS4/8 (A300-245A, Bethyl), PARP1 (ab32138, Abcam), GFP (ab5450, Abcam), PCNA (sc-56, Santa Cruz), KAP1 pS824 (A700-013, Bethyl), 6xHis (631212, Takara), V5 (A190-120A, Bethyl), ssDNA (autoanti-ssDNA, DSHB), dsDNA (autoanti-dsDNA, DSHB), PAN-ADPr (MABE1016, Merck), CST poly/Mono ADPr (83732, CST), Trevigen PAR monoclone (4335-MC-100, Trevigen), Millipore mono ADPr (MABE1076, Millipore), 10H ADPr (ALX-804-220-R100, Enzo Life Sciences). Secondary antibodies used here include: Alexa Fluor 488 donkey anti-goat, Alexa Fluor 555 donkey anti-rabbit, Alexa Fluor 555 donkey anti-mouse, Alexa Fluor 647 donkey anti-rabbit, Alexa Fluor 647 donkey anti-mouse (Thermo Fisher), goat anti-mouse HRP (P0447, Dako), goat anti-rabbit HRP (P0448, Dako). Autoanti-ssDNA and autoanti-dsDNA were deposited to the DSHB by Voss, E.W. (DSHB Hybridoma Product autoanti-ssDNA or autoanti-dsDNA).

### Clonogenic survival assay

U-2 OS cells were seeded at a density of 500 cells per well of a six-well plate and incubated for 24 h. Cells were then incubated with the desired condition for 14 days. Next, cells were washed in PBS and fixed using methanol with crystal violet (25% methanol, 0.5% crystal violet with distilled water) for 30 min. Following staining, cells were washed with water and imaged with a Nikon DSLR using identical exposure settings.

### Western blot

Whole cell extracts were obtained by lysing cells in 95°C SDS lysis buffer (1% SDS, 10 mM Tris, 1 mM EDTA, pH 8, 1 μM olaparib and 1 μM PARGi) and heating to 95°C for 3 min. Lysates were then cooled to RT and supplemented with 2 mM MgCl_2_ and 0.5 U/μl of benzonase and incubated at RT for 1–2 h before being heated to 95°C for 3 min and cooled to RT. Protein concentrations were then quantified using the standard Bradford assay (Bio-Rad, 5000006). Protein concentrations were then equalised and mixed with sample buffer (Thermo, NP0008). Proteins were then resolved by SDS-polyacrylamide gel electrophoresis (SDS-PAGE) and transferred onto nitrocellulose membranes. Membranes were blocked using 5% milk powder in PBS–Tween 20 (0.01%, hereafter PBST) for 1 h at RT. Membranes were briefly washed then probed for the indicated primary antibodies in 5% BSA in PBST at a dilution of 1:2500 at 4°C overnight. Membranes were then washed 3 × 5 min in PBST and subsequently incubated with the appropriate HRP-conjugated secondary antibodies (Dako) in 5% milk powder in PBST for 1 h. ECL-based chemiluminescence was detected using Pierce ECL (Thermo, 32106) with Hyperfilms (GE).

### Immunostaining

Cells were seeded onto 12 mm glass coverslips and grown to a density of ∼70–90% prior to treatment. Following the indicated treatments, cells were fixed in 3% formaldehyde for 15 min. For conditions where chromatin-bound RPA2, PCNA or ADP-ribose was examined, cells were pre-extracted with ice-cold 0.2% Triton X-100 in PBS for 1 min on ice prior to fixation. Following fixation, cells were washed 3× in PBS and then permeabilised using 0.2% Triton X-100 in PBS for 5 min. For experiments requiring PCNA labeling, permeabilisation was instead carried out using an ice-cold methanol:acetone solution (1:1) for 5 min following fixation. In both scenarios, cells were then washed 3× in PBS. When EdU was visualised, this was performed following the manufacturer's instructions prior to primary antibody incubation. Primary antibodies were incubated at a 1:500 dilution in DMEM supplemented with 10% FBS and penicillin-streptomycin antibiotics. Individual coverslips were incubated with 100 μl of DMEM containing the desired antibodies for 1–2 h at room temperature. Following primary antibody incubation, cells were washed 3× in PBS and secondary antibody incubations were performed as described for primary antibodies. Following antibody incubations, cells were washed 3× in PBS prior to incubation with 0.1 μg/ml DAPI in PBS for 10 minutes. Cells were then washed in 3× PBS before being washed in distilled water and air-dried on filter paper at RT. Dried coverslips were then mounted onto glass slides using 5 μl of Mowiol-based mounting media (Mowiol 4-88 [Sigma, 81381] in Glycerol/TRIS).

### Microscopy

Samples seeded were imaged using standard wide-field microscopy on a BX61 Olympus microscope equipped with 20×/0.5 and 40×/0.75 dry objectives, a CoolSNAP HQ2 14-bit detector (Roper Scientific), motorised stage (Prior) and MetaMorph 7.5 imaging software.

### Quantitative image based cytometry

Multichannel images for quantitative image based cytometry (QIBC) were were performed using the microscope described above. Typically, 25–50 non-overlapping images were randomly acquired with the 20× objective per condition to yield at least 1000 cells per sample. All images were acquired under non-saturating conditions at a single software autofocus directed *z*-position. For all conditions in one experiment identical settings were used.

Nuclei identification and segmentation was performed using two-class Otsu thresholding and watershed with CellProfiler (51). Identified nuclei objects were then used as a mask across all image channels and the pixel intensities for each channel were recorded and are depicted here as arbitrary units (A.U.). For subsequent analysis, nuclei were filtered to contain only interphase cells containing a 2C-4C DNA content as defined by total DAPI intensities. Coloured scatter plots and dot-bar plots were created using R. Within one experiment, similar numbers of identified nuclei were randomly sampled and compared across different conditions. For dot-bar plot visualisations, random x-axis jittering was applied to displace overlapping data points. All QIBC visualisations for each condition that contain at least 1000 cells are shown. CellProfiler pipelines and the R script used to generate QIBC plots are available at: https://github.com/callum-jpg/qibcPlot.

### DNA fiber spreading and analysis

In asynchronously growing cells, DarT expression (WT or E160A) was induced using doxycycline. Cells were then labeled with thymidine analogues 5-chloro-2′-deoxyuridine (CldU, 30 μM), washed 3 times with PBS, followed by 5-iodo-2′-deoxyuridine (IdU, 250 μM). The cells were then trypsinised and resuspended in ice-cold PBS at 2.5 × 10^5^ cells/ml. Labeled cells were then diluted 1:1 with unlabeled cells and 3 μl of these cells were mixed with 7.5 μl of lysis buffer (200 mM Tris–HCl pH 7.5, 50 mM EDTA, 0.5% (w/v) SDS) on a glass slide. After 9 min, the slides were tilted at 15°−45° and the resulting DNA spreads were air-dried and fixed in 3:1 methanol/acetic acid overnight at 4°C. The DNA fibers were denatured with 2.5 M HCl for 1 h, washed with PBS and blocked with 2% BSA in PBS supplemented with 0.1% Tween-20 for 40 min. The newly replicated CldU and IdU tracks were labeled (for 2.5 h in the dark, at RT) with anti-BrdU/CldU antibodies recognizing CldU (ab6326, Abcam, rat, 1:500) and BrdU/IdU (347580, Becton Dickinson, mouse, 1:100), respectively. After washing 5 × 3 mins in PBS supplemented with 0.2% Tween-20, the following secondary antibodies were used (incubated for 2 h in the dark, at RT): anti-mouse Alexa 488 (Molecular Probes, 1:300), anti-rat Cy3 (Jackson Immunoresearch, 1:150). After washing 5 × 3 mins each in PBS supplemented with 0.2% Tween-20 the slides were air-dried completely and mounted with 20 μl/slide ProLong Gold AntiFade (Thermo Fisher Scientific). Images were acquired using a Leica DM6 B fluorescence microscope equipped with a CCD camera (DMC 2900). CldU and IdU tract lengths were measured using the line tool in ImageJ software.

### Genomic DNA extraction

For U-2 OS gDNA extraction, cells were incubated with the indicated genotoxins and grown to 70–90% confluency. Cells were then trypsinised, pelleted at 800 × g for 3 min at 4°C, pellet gently washed with ice-cold PBS, pelleted again, before being resuspended in boiling lysis (1% SDS, 10 mM Tris, 1 mM EDTA, pH 8, 1 μM olaparib and 1 μM PARGi) buffer and heated to 95°C for 3 min. Lysates were then cooled to RT and treated with 20 μg/ml of RNase at 37°C for 1 h in a shaking incubator. Next, lysates were incubated with 100 μg/ml of proteinase K at 50°C for 1 h in a shaking incubator. Lysates were then cooled to RT and incubated with an equal volume of phenol:chloroform:isoamyl alcohol pH 8 (Sigma, P3803) and inverted until an emulsion formed. This emulsion was then centrifuged for 3 min at 15,000 × g and the upper aqueous layer was added to a fresh tube and re-extracted with fresh phenol:chloroform:isoamyl alcohol as before. This process was repeated until there was no white protein precipitate present at the interphase between the phenol and aqueous phases. Genomic DNA from the collected aqueous phase was then precipitated using 0.2 volumes of 10 M ammonium acetate and 2 volumes of 100% ethanol and inverted at RT until DNA precipitated. Genomic DNA was then pelleted at 5,000 × g for 3 min and the DNA pellet was washed twice with 70% ethanol before being resuspended in TE buffer. Genomic DNA concentrations were estimated using a DeNovix DS-11 FX nanodrop and concentrated by speecvac if required.

### ADP-ribosylated genomic DNA detection by dot blot

Approximately 1 μg of genomic DNA was dotted onto a nitrocellulose membrane (Amersham Protran 0.45 NC nitrocellulose) using a multichannel pipette, dried and then and crosslinked with 1200 J using a Stratalinker UV crosslinker. The crosslinked DNA was then immunoblotted for gDNA (autoanti-dsDNA, DSHB, 1:200) or ADP-ribose gDNA (Poly/Mono-ADP ribose, E6F6A, Cell Signalling Technology, 1:1000) for 1 h at RT in 5% powdered milk in PBS-T. Secondary peroxidase-couple antibodies (Dako) were incubated at RT for 1 h. ECL-based chemiluminescence was detected using Pierce ECL (Thermo, 32106) with Hyperfilms (GE).

### Counting motifs in gDNA

Non-overlapping motifs in genomic DNA were counted using the Python package motifSearch (available at: https://github.com/callum-jpg/motifSearch). The following bacterial species genomes were analysed: *T. aquaticus* (NZ_CP010822.1) and *E. coli* (NC_000913.3). For human autosomes, the GRCh38.p13 genome assembly was used.

## RESULTS

### TARG1 can reverse thymidine-linked ADP-ribosylation

**Figure 1.**
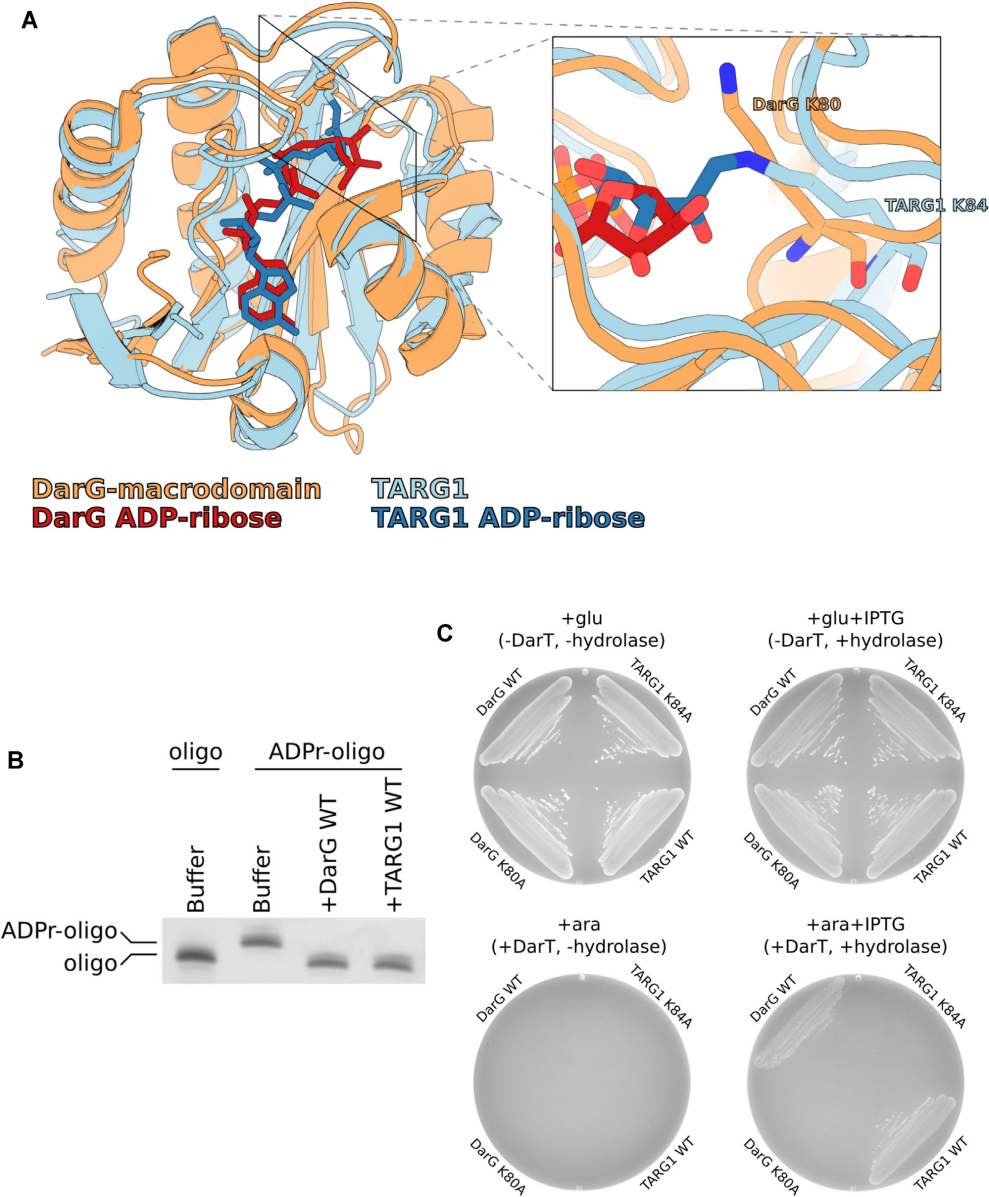
TARG1 removes thymidine-linked ADP-ribose from DNA and confers resistance to DarT toxicity in bacteria. (**A**) Structural comparison between TaqDarG-macrodomain (orange) bound to ADP-ribose (red) and TARG1 (light blue) with a covalent lysyl-ADP-ribose linkage (dark blue). To the right is a detailed view of the DarG catalytic lysine 80 (orange sticks) and TARG1 catalytic lysine 84 (blue sticks). (**B**) UV detection of DarT ADP-ribosylated DNA oligonucleotide de-ADP-ribosylation reactions with TaqDarG-macrodomain and TARG1. (**C**) Bacterial DarT toxicity rescue assay in BL21 DE3 using pBAD DarT and pET encoding DarG-macrodomain WT, DarG-macrodomain K80A, TARG1 WT or TARG1 K84A. pBAD expression is controlled with glucose or arabinose and pET expression is controlled with IPTG.

The initial paper which described the bacterial toxin DarT revealed that the macrodomain of the antitoxin DarG is structurally similar to that of TARG1, a human ADP-ribosylhydrolase, with an RMSD of 1.85 Å (Figure 1A) (30,38,42,44). This led us to explore the possibility that, like DarG, TARG1 could also reverse thymidine-linked ADP-ribosylation. To test this, we incubated a DarT ADP-ribosylated DNA oligonucleotide with either the DarG macrodomain or full length TARG1 recombinant proteins. The presence of either DarG or TARG1 resulted in loss of the thymidine-linked ADP-ribosylation from the DNA oligonucleotide (Figure 1B). Next, we determined whether the ADP-ribosylhydrolase activity of TARG1 could rescue DarT toxicity in bacteria. Co-expression of DarT with TARG1 revealed that TARG1 expression was able to rescue bacterial growth similarly to DarG. However, the equivalent catalytic lysine mutants K84A and K80A of TARG1 and DarG, respectively, were unable to rescue bacterial growth (Figure 1C). Collectively, these results show that, similar to DarG, TARG1 is able to reverse thymidine-linked ADP-ribosylation.

### TARG1-deficient cells are uniquely sensitive to DNA ADP-ribosylation

**Figure 2.**
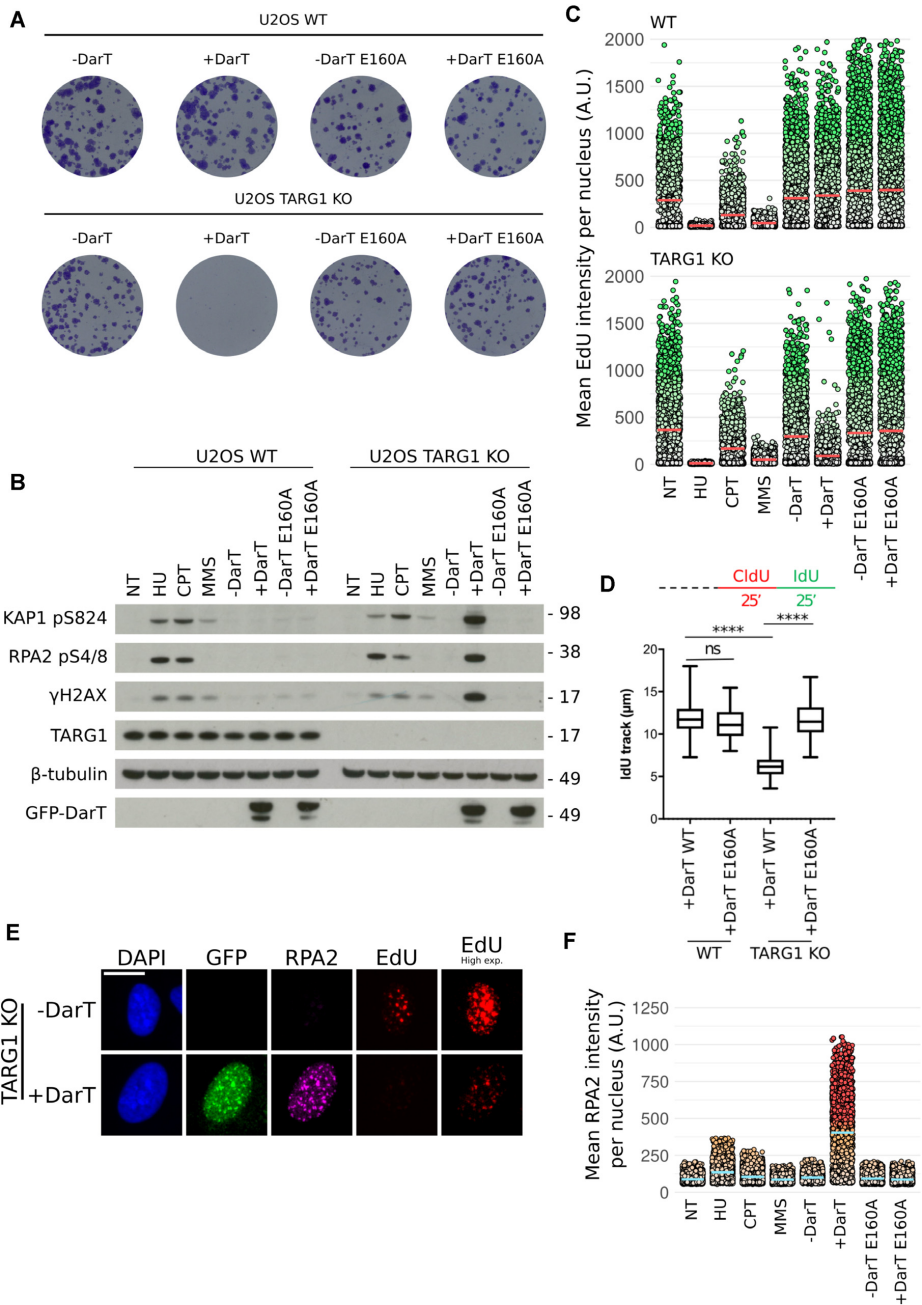
DarT induces the DDR in TARG1-deficient cells and limits DNA replication (**A**) Representative images of clonogenic survival in U-2 OS WT or TARG1 KO cells expressing pLIX_403 GFP-DarT WT or GFP-DarT E160A. pLIX_403 expression was controlled by doxycycline. (**B**) U-2 OS WT or TARG1 KO cells treated with HU (2 mM, 24 h), CPT (1 μM, 1 h), MMS (2 mM, 1 h), DarT WT (24 h), DarT E160A (24 h). Levels of DDR proteins KAP1 pS824, RPA2 pS4/8, γH2AX were analysed in response to the aforementioned genotoxins. (**C**) QIBC analysis of EdU incorporation in U-2 OS WT and TARG1 KO cells in response to the genotoxins found in Figure 2B. Prior to fixation, cells were treated with EdU (10 μM, 30 min). Red bar represents the mean EdU intensity for each population and each point represents the mean EdU intensity for an individual nuclei. (**D**) U-2 OS WT or TARG1 KO cells expressing either DarT WT or TARG1 KO for 24 h were pulse labeled with CldU followed by IdU for 25 min each, as indicated by the labeling protocol, top. IdU track lengths were measured for 100 fibers per condition. Statistical tests were performed using ANOVA where ns is not significant and **** is *P* < 0.0001. In the box plots, the box extends from the 25th (lower edge) to 75th (higher edge) percentiles and the line within the box represents the median. Whiskers extend to the minimum and maximum values recorded. Representative fiber images can be found in Supplementary Figure S2D. (**E**) U-2 OS TARG1 KO cells expressed GFP-DarT for 24 h and were treated with EdU (10 μM, 30 mins) prior to pre-extraction and fixation. Following a Click-iT reaction to visualise EdU, cells were then immunostained for GFP and chromatin-bound RPA2. Scale bar 20 μm. (**F**) QIBC analysis of chromatin-bound RPA2 of U-2 OS TARG1 KO cells in response to the genotoxins found in Figure 2B. Blue bar represents the mean RPA2 intensity for each population and each point represents the mean chromatin-bound RPA2 intensity for an individual nuclei.

In bacteria, the expression of DarT is extremely toxic due to the robust ADP-ribosylation of DNA (44). Due to TARG1 being a human ADP-ribosylhydrolase, we next began to ponder the impact DarT might have in human cells. In order to test this, we created a system for the doxycycline inducible expression of GFP-tagged full-length DarT wild-type (WT) from *Thermus aquaticus* or its catalytic mutant E160A in U-2 OS WT or TARG1 knockout (KO) cell lines. Interestingly, long-term clonogenic survival assays revealed that DarT WT expression had no apparent toxicity in WT cells. However, expression of DarT WT, but not E160A, in a TARG1 KO background was extremely toxic (Figure 2A). We next complemented TARG1 KO DarT WT and E160A cell lines with TARG1 WT or the TARG1 K84A catalytic mutant. Constitutive expression of TARG1 WT was able to rescue the cytotoxic effects of DarT, whereas TARG1 K84A was not (Supplementary Figure S1A). Together, these findings suggest that TARG1 loss renders cells uniquely sensitive to DarT-mediated thymidine-linked ADP-ribosylation of DNA.

Previous work has shown that expression of DarT in bacteria leads to induction of the SOS response, the bacterial DDR, as demonstrated by elevated levels of RecA (44–47). The striking toxicity of DarT in a TARG1 KO background led us to also examine the induction of the DDR in human cells as a result of this novel DNA adduct. To do so, we compared DarT as a genotoxin against a panel of well-characterised genotoxins, including: hydroxyurea (HU), a replication inhibitor; camptothecin (CPT), a DNA topoisomerase I poison; and methyl methanesulfonate (MMS), an alkylating agent that leads to base methylation and triggers ADP-ribosylation signaling mainly mediated by PARP1. In TARG1 KO cells, DarT WT, but not E160A, expression for 24h resulted in the accumulation of several DDR markers, namely phosphorylation of KAP1 (pS824), RPA2 (pS4/8) and H2AX (pS139, hereafter γH2AX) (Figure 2B). The DDR induced by DarT treatment was rescued by TARG1 WT, but not K84A, overexpression in TARG1 KO cells (Supplementary Figure S1B). Remarkably, despite strong induction of DNA damage signaling in TARG1 KO cells, no DDR markers were detected in WT cells expressing DarT. This suggests that the DNA damage inflicted by DarT is efficiently repaired by TARG1 that is present in WT cells. Although the magnitude of the DDR activation in response to these genotoxins is markedly different, we noted that the pattern of induction observed in DarT-expressing TARG1 KO cells is most similar to HU and CPT-treated cells (Figure 2B). Taken together, these results suggest that thymidine-linked ADP-ribosylation in TARG1 KO cells leads to robust DDR activation.

### DarT slows replication fork progression

**Figure 3.**
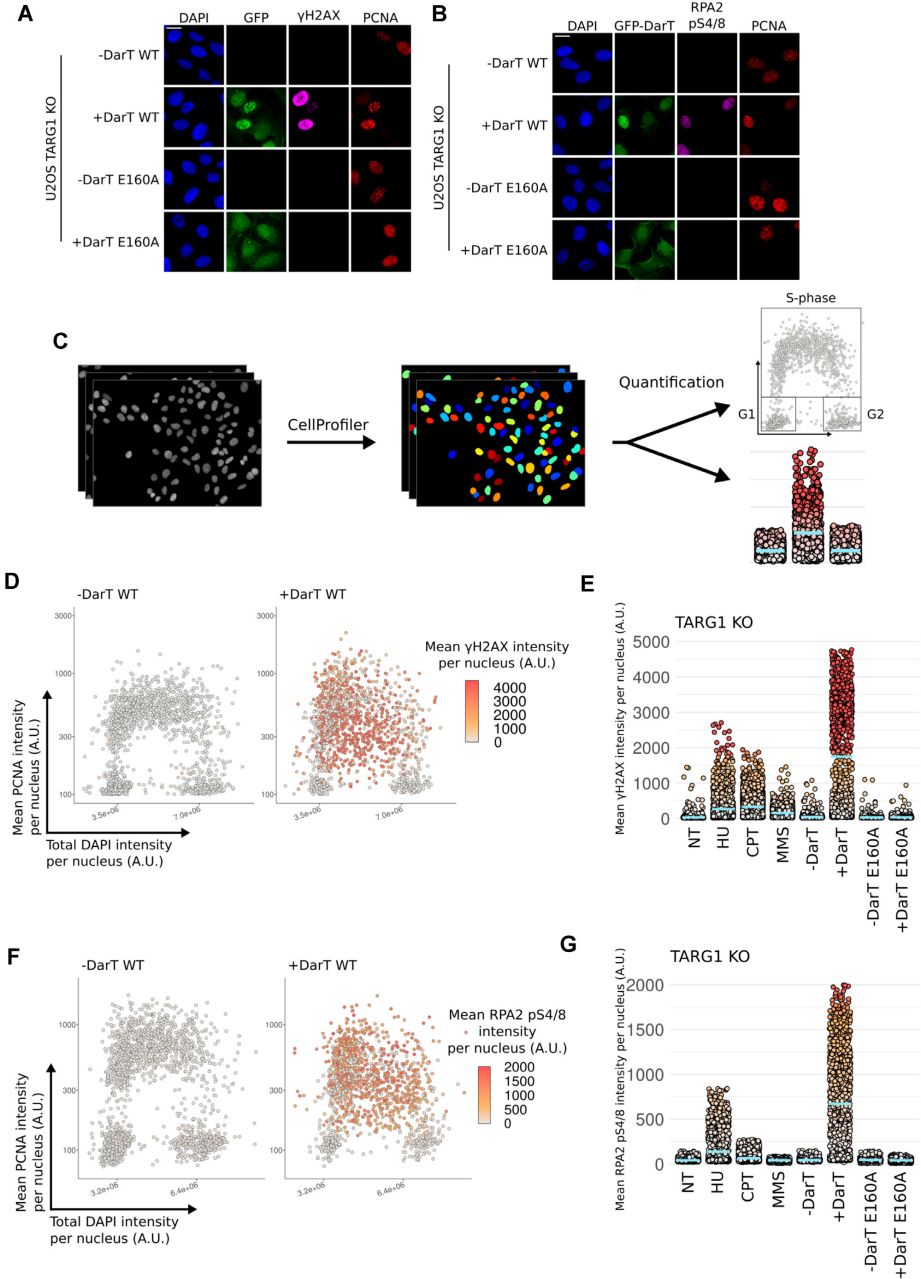
DarT induces the DDR at sites of DNA replication (**A**) Representative images of U-2 OS TARG1 KO cells expressing either GFP-DarT WT or E160A for 24 h were pre-extracted and immunostained for GFP, γH2AX and PCNA. Nuclear DNA was counterstained with DAPI. Scale bar 20 μm. (**B**) Representative images of U-2 OS TARG1 KO cells expressing either GFP-DarT WT or E160A for 24 h were pre-extracted and immunostained for GFP, RPA2 pS4/8 and PCNA. Nuclear DNA was counterstained with DAPI. Scale bar 20 μm. (**C**) Workflow for QIBC analysis. (**D**) QIBC analysis of asynchronous U-2 OS TARG1 KO cells expressing GFP-DarT WT for 24 h. Cells were pre-extracted, fixed and immunostained as in A. QIBC was used to record total DAPI intensity, mean PCNA intensity and mean γH2AX intensity per nucleus for >1000 cells. DAPI and PCNA intensities for individual cells were used to generate the scatter plot and γH2AX intensity was used to colour points. (**E**) QIBC analysis of γH2AX in TARG1 KO cells in response to the genotoxin treatments found in Figure 2B. Blue bar represents the mean γH2AX intensity for each population and each point represents the mean γH2AX intensity for an individual nuclei. (**F**) U-2 OS TARG1 KO cells were treated and analysed as in (D) and immunostained as in (B). (**G**) QIBC analysis as in E and immunostained as in (B).

Since HU, CPT, MMS (in human cells) and DarT (in bacteria) have previously been shown to impair DNA synthesis, we next compared EdU incorporation for all of these genotoxins in human cells (44,45,52). To investigate this, we used automated microscopy, CellProfiler and R to perform quantitative image based cytometry (QIBC) as shown in Figure 3C (51). Analysis of EdU pixel intensities for single cells in microscopy images revealed that HU, CPT and MMS impact EdU incorporation similarly between WT and TARG1 KO cells. However, DarT WT, but not E160A, specifically limits EdU incorporation in TARG1 KO cells with no impact on WT cells (Figure 2C. Immunofluorescence images: Supplementary Figure S2A. Further QIBC: Supplementary Figure S2B). Interestingly, EdU incorporation is not ablated in DarT-treated TARG1 KO cells, unlike those treated with HU, suggesting that some DNA synthesis is able to take place. Assuming that TNTC is the only DNA sequence ADP-ribosylated by DarT, this motif has a maximum occurrence of 1.8% in human autosomal DNA (Supplementary Figure S2C). This suggests that there will exist regions of DNA in which replication can proceed unperturbed by DarT. The impact on total DNA replication prompted us to examine replication forks at a single-molecule level by using a DNA fiber spreading assay. Pulse labeling with CldU followed by IdU of U-2 OS WT or TARG1 KO cells expressing either DarT WT or E160A for 24h revealed that DarT markedly slows down, but does not completely block, replication fork progression in TARG1 KO cells, with no impact observed in WT cells (Figure 2D. Representative fibers: Supplementary Figure S2D). Indeed, by using immunofluorescence, low levels of DNA synthesis in DarT-treated TARG1 KO cells were visible as EdU foci that co-localise with sites of chromatin-bound RPA2, a marker of ssDNA. Interestingly, GFP-tagged DarT also forms distinct foci in a subset of cells that co-localise with both EdU incorporation and chromatin-bound RPA2 (Figure 2E). QIBC analysis of chromatin-bound RPA2 further revealed extensive ssDNA generation in DarT-treated cells (Figure 2F). This suggests that DarT strongly targets sites of active DNA replication in order to ADP-ribosylate its substrate ssDNA, which in turn impedes replication fork progression.

### DarT induces the DDR at sites of DNA replication

Due to the co-localisation of DarT and chromatin-bound RPA2 with sites of DNA replication, we next examined if different DNA damage markers also followed the same pattern. Due to DarT limiting EdU incorporation, we used chromatin-bound PCNA as a DNA synthesis-independent marker for sites of DNA replication and thus for identifying cells in S-phase. We observed that γH2AX formation occurs in PCNA-positive cells, with a subset of cells showing strong pan-nuclear γH2AX signal, indicating extensive DNA breakage (Figure 3A). We also observed that some cells form distinct γH2AX foci which co-localise with DarT and PCNA, perhaps representing an early stage of DNA breakage caused by DarT that progressively spreads to form the pan-nuclear γH2AX signal. Similarly, DarT expression also leads to the phosphorylation of RPA2 at S4/8 in PCNA-positive cells (Figure 3B). Treatment of cells with either HU or CPT also led to induction of γH2AX or RPA2 pS4/8 in PCNA-positive cells, albeit to a lesser extent (Supplementary Figure S3A, B). We next quantified microscopy images for these conditions using QIBC to better determine in which stage of the cell cycle these DNA damage markers were induced. This further revealed that the observed induction of γH2AX or RPA2 pS4/8 occurs within PCNA-positive cells (Figure 3D, F). Similarly, HU and CPT treatments also induced γH2AX and RPA2 pS4/8 in PCNA-positive cells (Supplementary Figure S4A, B). The lower levels of γH2AX or RPA2 pS4/8 recorded by QIBC following genotoxin treatment were consistent with detection of the same markers by western blot (Figure 3E, G). Taken together, this suggests that DarT treatment in TARG1 KO cells leads to extensive activation of the DDR in S-phase.

### DarT induces ADP-ribosylation at ssDNA sites during DNA replication

**Figure 4.**
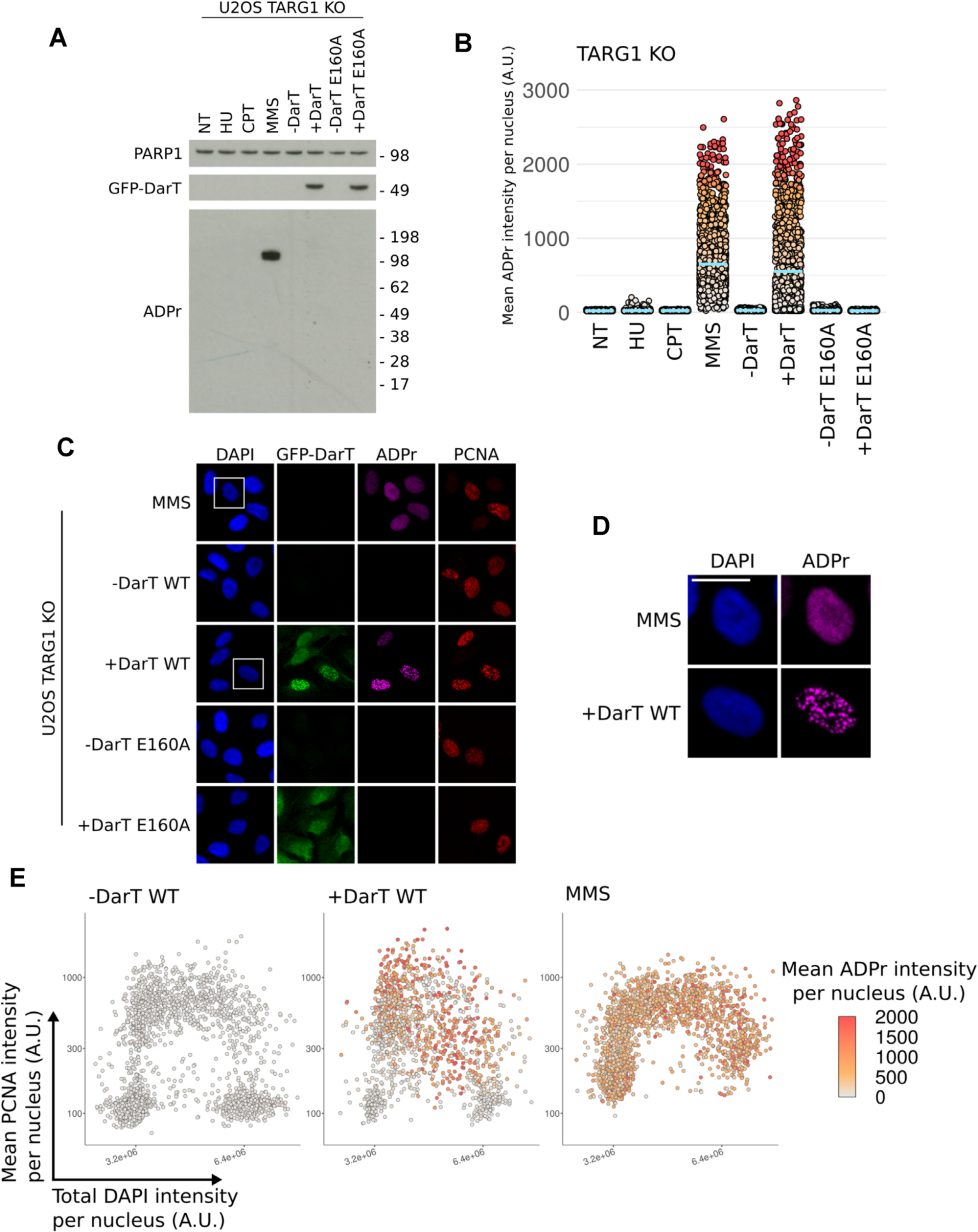
DarT induces ADP-ribose foci in S-phase cells with no ADP-ribosylation detectable by western blot. (**A**) U-2 OS TARG1 KO cells treated with HU (2 mM, 24 h), CPT (1 μM, 1 h), MMS (2 mM, 1 h), DarT (24 h), DarT E160A (24 h). Levels of ADP-ribosylation were assessed using the CST poly/mono ADP-ribose antibody in response to the aforementioned genotoxins. (**B**) QIBC analysis of ADP-ribosylation in TARG1 KO cells in response to the genotoxin treatments in A. Blue bar represents the mean ADP-ribosylation intensity for each population and each point represents the mean ADP-ribosylation intensity for an individual nuclei. (**C**) Representative images of U-2 OS TARG1 KO cells expressing either GFP-DarT WT or E160A for 24 h or cells treated with MMS (2 mM, 1 h) were pre-extracted and immunostained for GFP, ADP-ribosylation, PCNA and nuclear DNA was counterstained with DAPI. Scale bar 20 μm. (**D**) Magnified view of the region enclosed by the white square in (B) for DarT and MMS-treated cells. Scale bar 20 μM. (**E**) QIBC analysis of asynchronous U-2 OS TARG1 KO cells expressing GFP-DarT WT for 24 h or treated with MMS (2 mM, 1 h). Cells were pre-extracted, fixed and immunostained as in (C). QIBC was used to record total DAPI intensity, mean PCNA intensity and mean ADP-ribosylation intensity per nucleus for >1000 cells. DAPI and PCNA intensities for individual cells were used to generate the scatter plot and ADP-ribosylation intensity was used to color points.

Since DarT is an ART, we next examined the levels of protein ADP-ribosylation observed in response to this genotoxin. Remarkably, despite leading to a strong induction of the DDR, no protein ADP-ribosylation was detected in DarT-treated cells when analysed by western blot (Figure 4A). This contrasts the strong PARP1 auto-modification observed in MMS-treated cells, which is activated by the SSB repair pathway in order to repair DNA alkylation (4). Curiously, QIBC analysis using the same ADP-ribose recognising antibody (CST poly/mono ADP-ribose) revealed that cells treated with either DarT or MMS both show increased ADP-ribosylation signaling, suggesting that DarT-mediated ADP-ribosylation cannot be detected by western blot (Figure 4B). Further analysis of ADP-ribosylation induced by DarT shows that this signal occurs within PCNA-positive cells and forms distinct foci at sites of DNA replication (Figure 4C–E, Supplementary Figure S5A, B). Contrasting this, ADP-ribosylation induced by MMS forms indiscriminately of the cell cycle stage and shows a different foci pattern. Taken together, these results suggest that ssDNA exposed during S-phase is ADP-ribosylated by DarT. Furthermore, these data also suggest that the ADP-ribosylation signal we observe in DarT-treated cells is found on nucleic acids, as indicated by the lack of detectable protein ADP-ribosylation by western blot.

**Figure 5.**
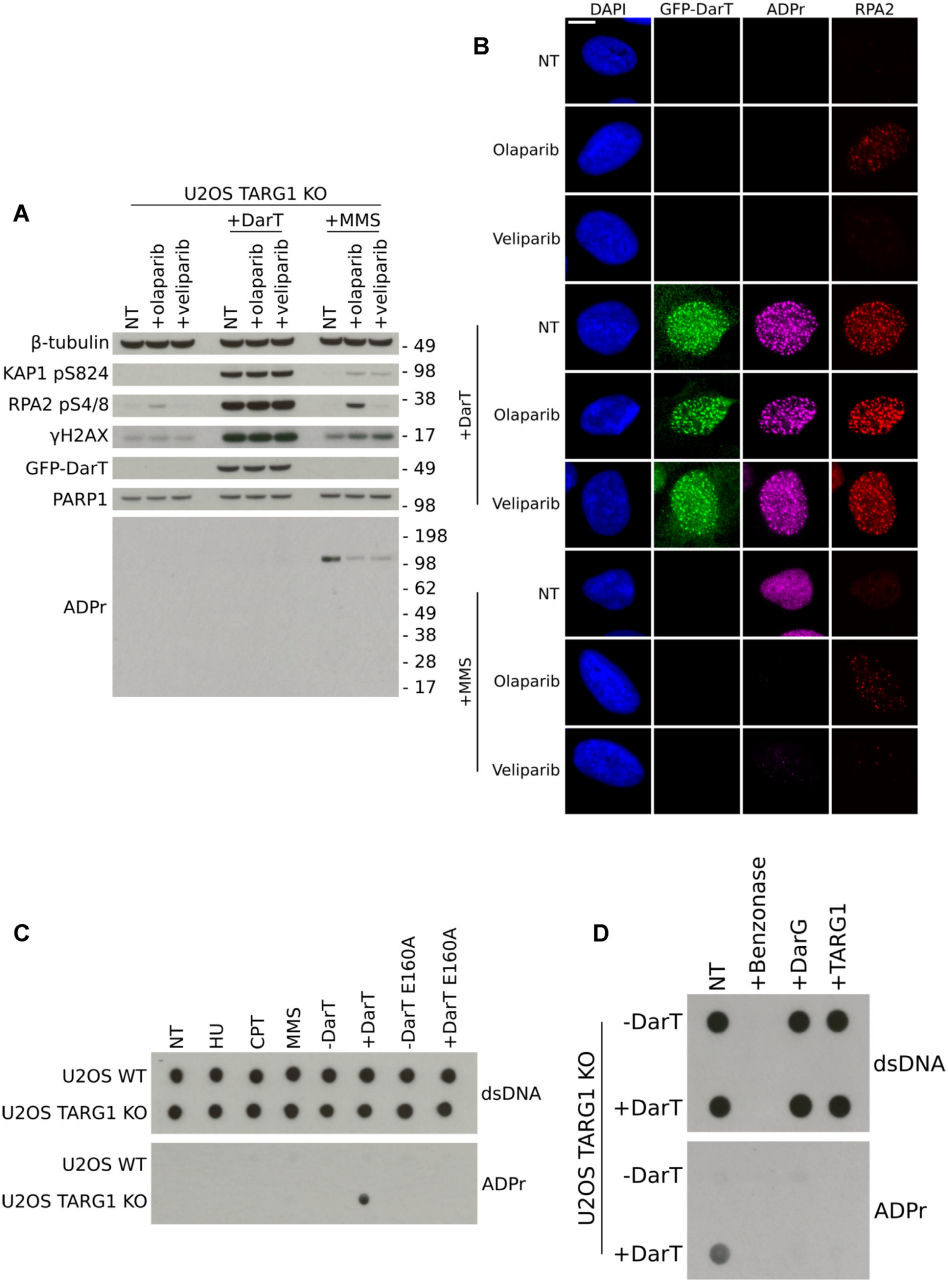
Detection of thymidine-linked ADP-ribosylation in human genomic DNA. (**A**) U-2 OS TARG1 KO cells expressing GFP-DarT (24 h) or treated with MMS (2 mM, 1 h) were also treated with olaparib (10 μM, 24 h) or veliparib (10 μM 24 h). Levels of KAP1 pS824, RPA2 pS4/8, γH2AX and ADP-ribosylation were assessed. (**B**) Representative images of U-2 OS TARG1 KO cells treated as in (A). Cells were pre-extracted and immunostained for GFP, ADP-ribosylation and chromatin-bound RPA2. Scale bar 10 μm. (**C**) U-2 OS WT or TARG1 KO cells treated with HU (2 mM, 24 h), CPT (1 μM, 1 h), MMS (2 mM, 1 h), DarT WT (24 h), DarT E160A (24 h). Genomic DNA was extracted and dotted onto nitrocellulose membranes and immunoblotted for dsDNA or ADP-ribosylation using the CST poly/mono ADP-ribose antibody. (**D**) Genomic DNA from U-2 OS TARG1 KO cells expressing DarT was extracted as in (C). DNA was incubated with either 1 μM TaqDarG-macrodomain WT or TARG1 WT recombinant proteins in vitro. DNA was then immunoblotted as in (C).

DarT is a mono-ADP-ribosyltransferase, which suggests that the ADP-ribosylation observed in DarT-treated cells is in the mono form. However, it is conceivable that PARPs, such as PARP1, could elongate mono-ADP-ribosylation to form poly-ADP-ribosylation, an event which has previously been shown to occur on DNA end phosphates *in vitro* (40). To examine this, we first determined if PARP inhibitors could inhibit the ART activity of DarT. Incubation of DarT with DNA and olaparib or veliparib *in vitro* did not lead to inhibition of DarT activity (Supplementary Figure S6). DarT-treated cells incubated with either olaparib or veliparib *in vivo* also had no observable change in DDR markers KAP1 pS824, RPA2 pS4/8 or γH2AX, whereas MMS-treated cells experienced a reduction in PARP1 auto-modification in response to these inhibitors (Figure 5A). We next examined by immunofluorescence if any change in the ADP-ribosylation signal could be detected in PARP inhibitor-treated cells. In response to DarT treatment in TARG1 KO cells there was no detectable difference in ADP-ribosylation, whereas in MMS-treated cells the ADP-ribosylation signal was lost (Figure 5B). For cells exhibiting GFP-DarT foci, these foci co-localise with both ADP-ribosylation and chromatin-bound RPA2 foci, demonstrating that the ADP-ribosylation we observe is at sites of ssDNA (Figure 5B). These results reveal that a majority of DarT-mediated DNA ADP-ribosylation is not extended to the poly form by PARPs and is likely present in the mono-ADP-ribose form, since PARP inhibition does not impact the ADP-ribosylation signal observed by immunofluorescence.

### Detection of ADP-ribosylated human genomic DNA

Previous work has shown that DarT only targets ssDNA, with no activity towards macromolecules such as RNA or protein. Due to the observation that the ADP-ribosylation induced by DarT was only detectable by immunofluorescence and not by western blot when using the CST poly/mono ADP-ribose antibody, we reasoned this antibody was able to recognise DNA ADP-ribosylation. We therefore compared the CST poly/mono ADP-ribose antibody alongside a panel of other ADP-ribose binding reagents against either an unmodified or DarT ADP-ribosylated DNA oligonucleotide. We confirmed that only the CST poly/mono ADP-ribose antibody and, to some extent, the PAN-ADP-ribose reagent can recognise this DNA modification (Supplementary Figure S7). Following the serendipitous discovery that the CST poly/mono ADP-ribose antibody specifically recognises thymidine-linked ADP-ribosylation of DNA with no cross-reactivity to unmodified DNA, we purified gDNA from human cells in order to confirm detection of *in vivo* DNA ADP-ribosylation. For all genotoxins tested, only DarT-treated TARG1 KO cells showed detectable gDNA ADP-ribosylation when analysed by dot blot (Figure 5C). Furthermore, *in vivo* ADP-ribosylated gDNA was reversed by the ADP-ribosylhydrolases DarG or TARG1 *in vitro* and both DNA and ADP-ribosylation signals were lost following benzonase treatment (Figure 5D). Collectively, these data demonstrate that *in vivo* ADP-ribosylation of gDNA from human cells is detectable by dot blot using the CST poly/mono ADP-ribose antibody, providing the first detection of reversible DNA ADP-ribosylation *in vivo* in human cells.

## DISCUSSION

ADP-ribosylation is a dynamic modification involved in a vast array of cellular processes such as the DDR, chromatin remodeling, transcription and cell death (24,53,54). In these processes, ADP-ribosylation has been primarily studied as a post-translational modification of proteins. However, growing evidence demonstrates that nucleic acids can also be targets for ADP-ribosylation. For example, the roles of PARP1-3 in DNA damage repair are extensively characterised through their auto-modification and ADP-ribosylation of target proteins, but recently PARP1-3 have also been shown to ADP-ribosylate phosphate groups at DNA ends *in vitro* (37–41).

However, examination of the *in vivo* impact of nucleic acid ADP-ribosylation in human cells has been scarcely studied (55,56). Here, our results reveal the first detection of an ADP-ribose DNA adduct on thymidine following expression of the genotoxin DarT in human cells. We also demonstrate that this base adduct is reversed by TARG1, similar to what was shown in bacteria with DarG (44). The catalytic activity of TARG1 in reversing thymidine-linked ADP-ribosylation also appears to be non-redundant with other hydrolases. This contrasts the finding *in vitro* that TARG1, MacroD2, ARH3 and PARG can reverse DNA terminal phosphate ADP-ribosylation, further highlighting the unique and strikingly specific catalytic activity of TARG1 (38). Moreover, we also show the significant impact that unchecked DNA ADP-ribosylation can impose on cells that are deficient in the ADP-ribosylhydrolase TARG1. We demonstrate that the genotoxin DarT induces DNA ADP-ribosylation stress that results in robust activation of the DDR in replicating cells and slows replication fork progression. We conclude that TARG1 repairs and thereby protects ssDNA exposed during DNA replication from the damaging impact of bulky ADP-ribose base adducts. Replication stress induced by these bulky ADP-ribose adducts also yields potential avenues for future research. DarT could be utilised as an easy to control tool that can specifically stall replisomes at bulky ADP-ribose adducts in specific regions of the genome. This could reveal additional DDR factors involved in overcoming this form of damage.

We note that the action of TARG1 in the reversal of thymidine-linked ADP-ribose adducts is analogous to direct DNA damage repair pathways that reverse DNA damage without requiring breakage of the DNA phosphodiester backbone, DNA synthesis or a homologous nucleotide template (14,15). Furthermore, the activity of TARG1 is also somewhat similar to the DNA repair enzyme aprataxin, which removes AMP adducts from the 5′-phosphates of DNA (57). Moreover, the ADP-ribosylhydrolases TARG1, PARG, MacroD2 and ARH3 have also been shown to reverse phosphate-linked DNA ADP-ribosylation *in vitro* (38). We therefore propose TARG1 as a non-canonical direct DNA repair enzyme involved in the removal of ADP-ribose adducts from DNA, similar to what was shown for DarG in bacteria (44–46).

Here, we demonstrate the first detection of reversible ADP-ribosylated human gDNA *in vivo*, forming a proof-of-concept methodology for the detection of cellular DNA ADP-ribosylation. Furthermore, we highlight important considerations in the detection of DNA ADP-ribosylation. Namely, the detection of a strong ADP-ribosylation signal by immunofluorescence but no discernible ADP-ribosylation detected by western blot reveals the potentially hidden nature of this nucleic acid modification in cells. This has significant implications for future studies examining ADP-ribosylation, indicating that the presence of nucleic acid ADP-ribosylation can easily be missed by traditional techniques or misinterpreted as protein modification. With this in mind, the delay in the study of nucleic acid ADP-ribosylation when compared to its protein counterpart could be explained by the similarity of ADP-ribosylation to nucleic acids and the potential lability of the linkage between ADP-ribose and nucleic acids, both of which could make this modification difficult to detect. We envisage that future work will develop more sensitive methods to study this modification in order to extend *in vitro* observations of PARP-mediated nucleic acid ADP-ribosylation into an *in vivo* context.

The reversal of thymidine-linked ADP-ribosylation by TARG1, shown here, and previous work demonstrating that both DNA and RNA end ADP-ribosylation can be reversed by TARG1 led us to question the potential physiological role of this ADP-ribosylhydrolase (38,42). Previous work has shown that a TARG1 homozygous mutation in patients led to neurodegenerative disease but the protein substrates for TARG1 are not clear (30). Perhaps TARG1 has a role in protecting against neurodegeneration by repairing nucleic acid ADP-ribose adducts. Neurodegeneration develops as a result of abnormalities in neuronal and non-neuronal cells in the nervous system (58). However, neuronal cells are typically quiescent, suggesting that their post-replicative nature would not be susceptible to DNA adducts catalysed by an ART with similar activity to DarT. However, thymidine ADP-ribosylation could arise from a previous event, such as neuronal differentiation, that would persist in TARG1-deficient cells. Alternatively, the finding that TARG1 can reverse ADP-ribosylation on phosphates at both DNA and RNA ends may suggest that other forms of nucleic acid metabolism, such as transcription, could be susceptible to nucleic acid ADP-ribose adducts (38,42). Non-neuronal cells of the nervous system, such as glia, can be replicative and therefore a DarT-like ART could modify ssDNA during DNA replication. A failure of TARG1 to repair nucleic acid ADP-ribose adducts in neuronal and non-neuronal cells could contribute to the development of neuropathological symptoms. We hope that the development of more sensitive methods for the detection of nucleic acid ADP-ribosylation will enable future research to assess if nucleic acid ADP-ribosylation is enriched in TARG1-deficient patient cells and if this modification can be used as a biomarker for disease.

However, there is currently no known ART in human cells that can ADP-ribosylate thymidine bases and the endogenous form of this modification has not yet been detected. Here, we have created an artificial system with DarT to examine the impact that extreme DNA ADP-ribosylation can have. Whether an ART with similar catalytic activity to DarT exists in human cells remains to be determined. Nonetheless, the ART superfamily is renowned for being tremendously diverged at the sequence level, making the prediction of new ARTs difficult and new diverged PARP-like proteins are still being discovered (19,59). DarT was initially studied due to its presence in an operon with another protein containing a conserved macrodomain, DarG. DarT was then serendipitously characterised as a ssDNA-specific ART. This reveals the still unexpected nature of ARTs and highlights the requirement for the further characterisation of these enzymes across a range of substrates. Furthermore, the discovery of the PARP1-2 cofactor HPF1 has also recently revealed that the substrate specificity of PARP1-2 can be shifted towards serine in a DNA damage context, suggesting that other cofactors that alter substrate specificity, perhaps to nucleic acids, may also exist (25,26,60). This further highlights the diverse and changeable nature of ARTs and the requirement for additional methods to specifically detect different types of ADP-ribosylation.

Our discovery of the striking sensitivity of TARG1-deficient cells to DarT mediated DNA ADP-ribosylation with no impact on WT cells also yields potential avenues for cancer therapy. The cBioPortal curation of cancer genomic databases reveals that TARG1 is altered in approximately 1% of cancers (61). Albeit a rare mutation, the subset of cancers deficient in TARG1 catalytic activity could be specifically targeted for precise therapy by the genotoxin DarT. Bacterial toxins conjugated with proteins that bind an overexpressed cell receptor have been previously developed and used in anti-cancer therapy. For example, diphtheria toxin, which ADP-ribosylates eEF2, conjugated to interleukin-2 (ONTAK) has been approved by the FDA for the treatment of T-cell lymphomas (62,63). Furthermore, recent work has shown that TccC3 from the insect pathogen *Photorhabdus luminescence*, which ADP-ribosylates actin, can be conjugated to a modified form of the protective antigen (PA) from *Bacillus anthracis* and specifically guided to cancer cells overexpressing HER2 or EGFR receptors (64). PA forms an oligomeric pore following receptor binding and can facilitate the transport of a range of heterologous proteins into the cytosol of target cells (65–68). Similar strategies could be explored for the targetted killing of TARG1-deficient cancer cells by the genotoxin DarT with minimal side effects.

Futhermore, an intriguing pathophysiological possibility could be that DarT is secreted as an effector toxin by bacteria into host cells during infection. In this context, TARG1 would possess a protective function against DarT-like effector toxins. Thousands of currently sequenced bacterial genomes, including global pathogens, contain DarT-like sequences and it is therefore possible that a DarT orthologue is used as an effector toxin during bacterial infection. Analogously, cholera toxin from *Vibrio cholerae* once inside host cells mono-ADP-ribosylates the catalytic arginine of the alpha subunit of G-proteins, which subsequently leads to constitutive activation of the host adenylate cyclase (69,70). Under these conditions, the ADP-ribosylhydrolase ARH1 has a protective function by reversing cholera toxin induced arginine-linked ADP-ribosylation (71). Therefore TARG1 may possess a similar role in defending against toxic DNA ADP-ribosylation during infection.

Here, we have demonstrated that TARG1 is a non-canonical DNA repair enzyme that directly removes an ADP-ribose DNA base adduct. We have also shown that TARG1-deficient cells are uniquely sensitive to DNA ADP-ribosylation, which leads to a robust activation of the DDR in replicating cells. It would be exciting to see if endogenous thymidine-linked ADP-ribosylation or a similar adduct exists in human cells. Moreover, by using DarT as a tool to induce DNA ADP-ribosylation stress, we reveal the techniques and considerations required to detect this modification. With growing *in vitro* evidence revealing that PARPs can also ADP-ribosylate nucleic acids, the techniques demonstrated here may be further used and developed to study a new and exciting facet of ADP-ribosylation signalling.

## DATA AVAILABILITY

Plasmids and cell lines are available upon request. Code used to generate QIBC plots can be found at the GitHub repository https://github.com/callum-jpg/qibcPlot. Code used to predict genomic DNA motif frequency can be found at the GitHub repository https://github.com/callum-jpg/motifSearch.

## Supplementary Material

gkab771_Supplemental_File
