# Supplementary material for: TARG1 protects against toxic DNA ADP-ribosylation
Source: Nucleic Acids Res. 2021 Sep 11;49(18):10477–92. doi: 10.1093/nar/gkab771 (PMC8501950; doi:10.1093/nar/gkab771)
Supplement: gkab771_Supplemental_File [file gkab771_supplemental_file.pdf]

TARG1 protects against toxic DNA ADP-ribosylation

Callum Tromans-Coia, Andrea Sanchi, Giuliana K. Moeller, Gyula Timinszky, Massimo Lopes, Ivan Ahel

Supplementary Information

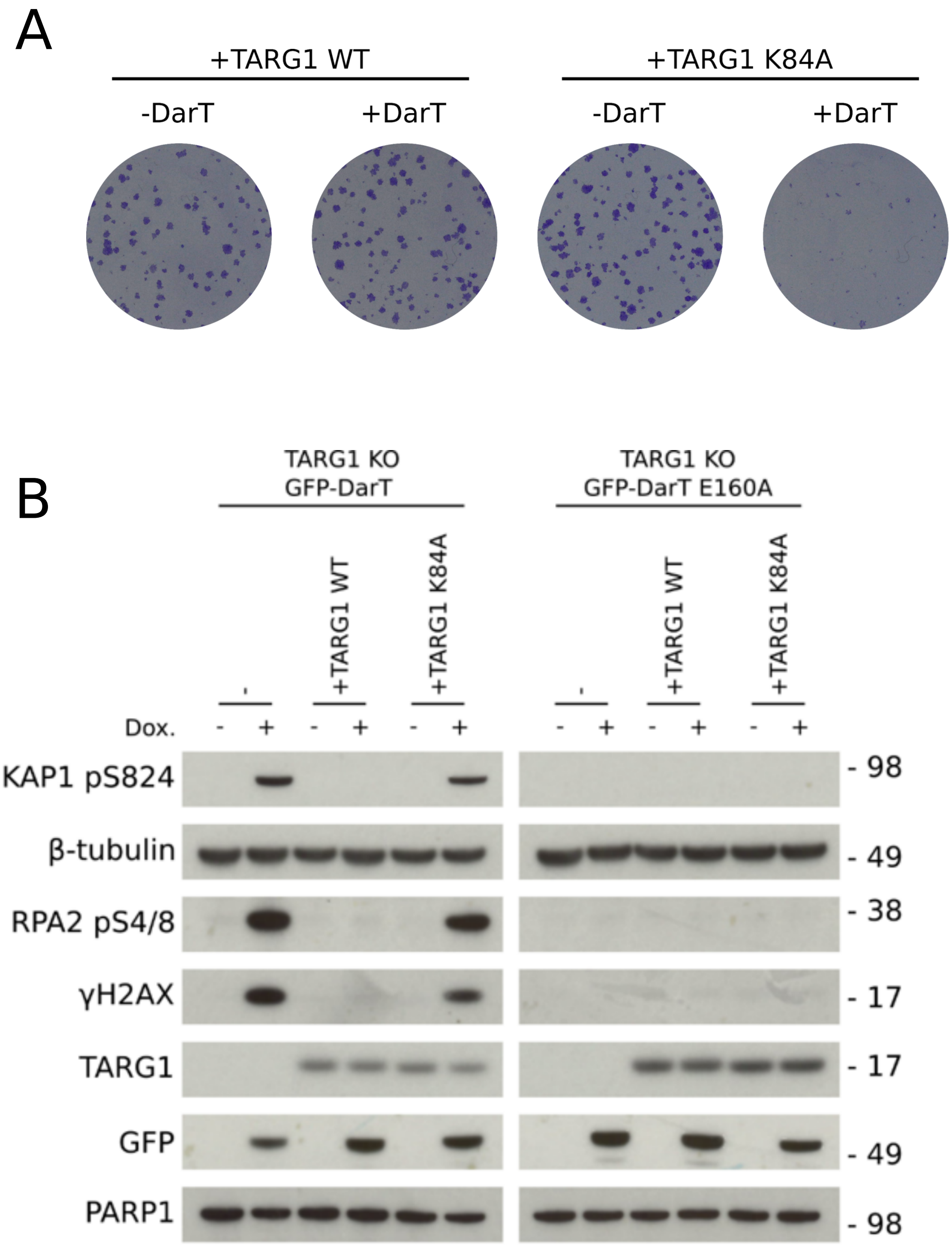

Figure S1
TARG1 overexpression rescues DarT toxicity in human cells.
A - Representative images of clonogenic survival in U-2 OS TARG1 KO cells expressing DarT WT (24h) in the presence of constitutively expressed pLX304 TARG1 WT or TARG1 K84A.
B - U-2 OS TARG1 KO cells expressing either GFP-DarT WT or E160A (24h) in combination with constitutively expressed pLX304 TARG1 WT or K84A. Levels of DDR proteins KAP1 pS824, RPA2 pS4/8 and γH2AX were assessed.

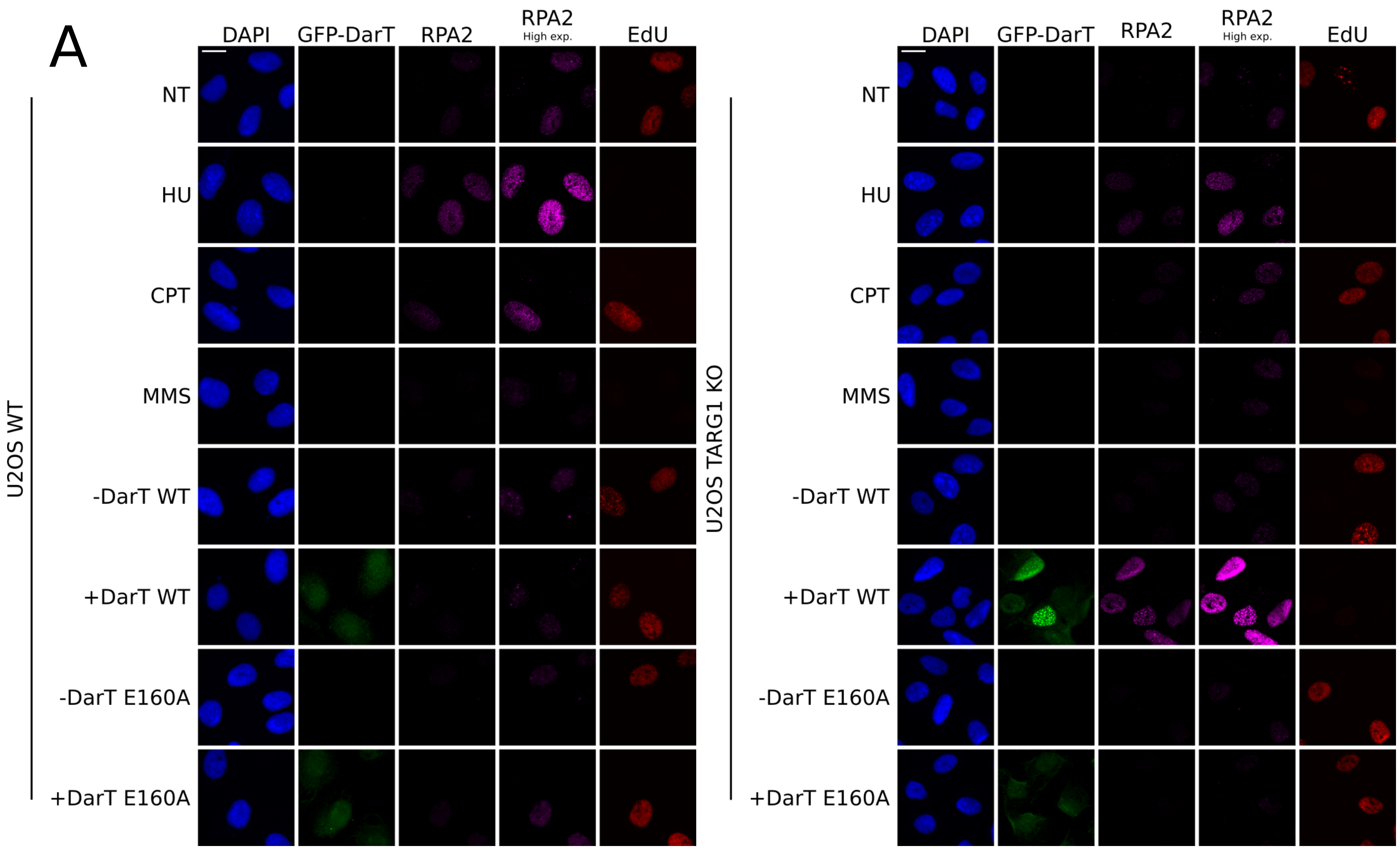

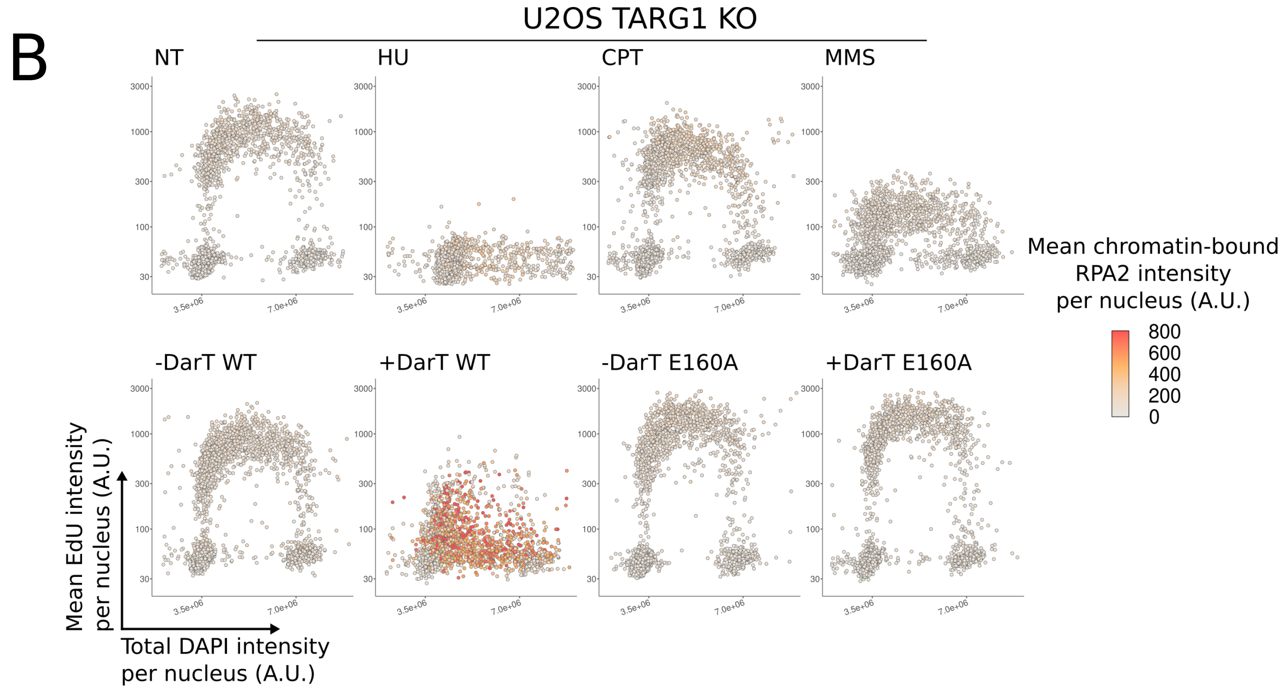

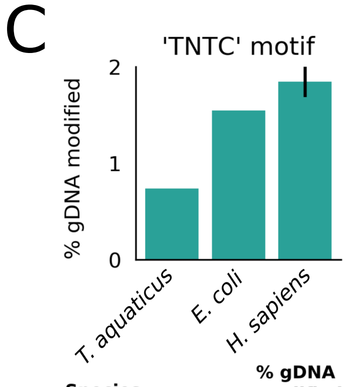

Species	% gDNA modified
Thermus aquaticus	0.74
Escherichia coli	1.55
Homo sapiens	1.85±0.16

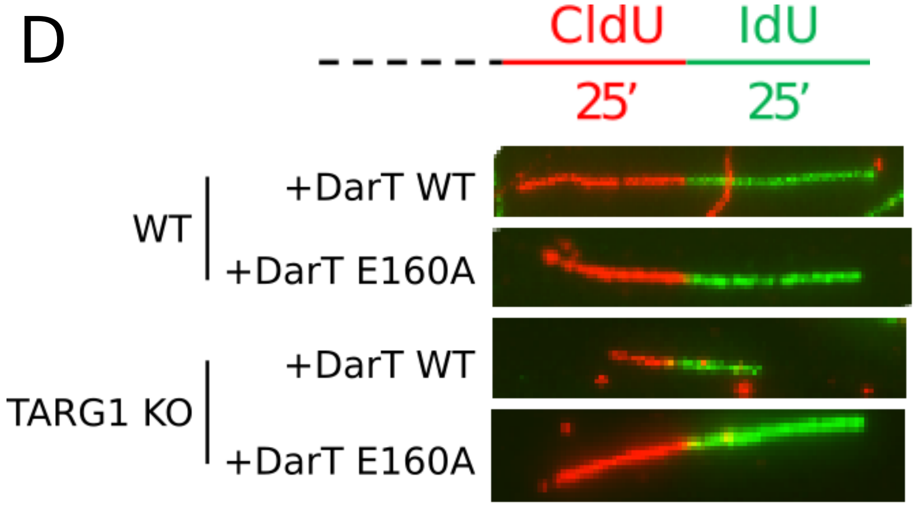

Figure S2
DarT limits EdU incorporation in TARG1 KO cells.
A - Extended representative images of both U-2 OS WT and TARG1 KO found in Figure 2E with genotoxin treatments described in Figure 2B.
B - QIBC analysis of asynchronous U-2 OS TARG1 KO cells treated with genotoxins described in Figure 2B. Cells were incubated with EdU (10 µM, 30 mins), pre-extracted, fixed and immunostained as described in Figure 2E. QIBC was used to record total DAPI intensity, mean EdU intensity and mean chromatin-bound RPA2 intensity per nucleus for >1,000 cells. DAPI and EdU intensities for individual cells were used to generate the scatter plot and chromatin-bound RPA2 intensity was used to colour points.
C - Bioinformatic search for the theoretical maximum number of non-overlapping TNTC DNA motifs on ssDNA which could me modified by DarT for the indicated genome. Values represent the percent-age of total genomic DNA which contained a TNTC motif. For *H. sapiens* only the autosomes were analysed with SD displayed.
D - DNA fibers following DarT WT or E160A (24h) treatment. CldU or IdU containing tracts were immunostained red or green, respectively. A single representative fiber for each condition is shown with the labelling protocol, top.

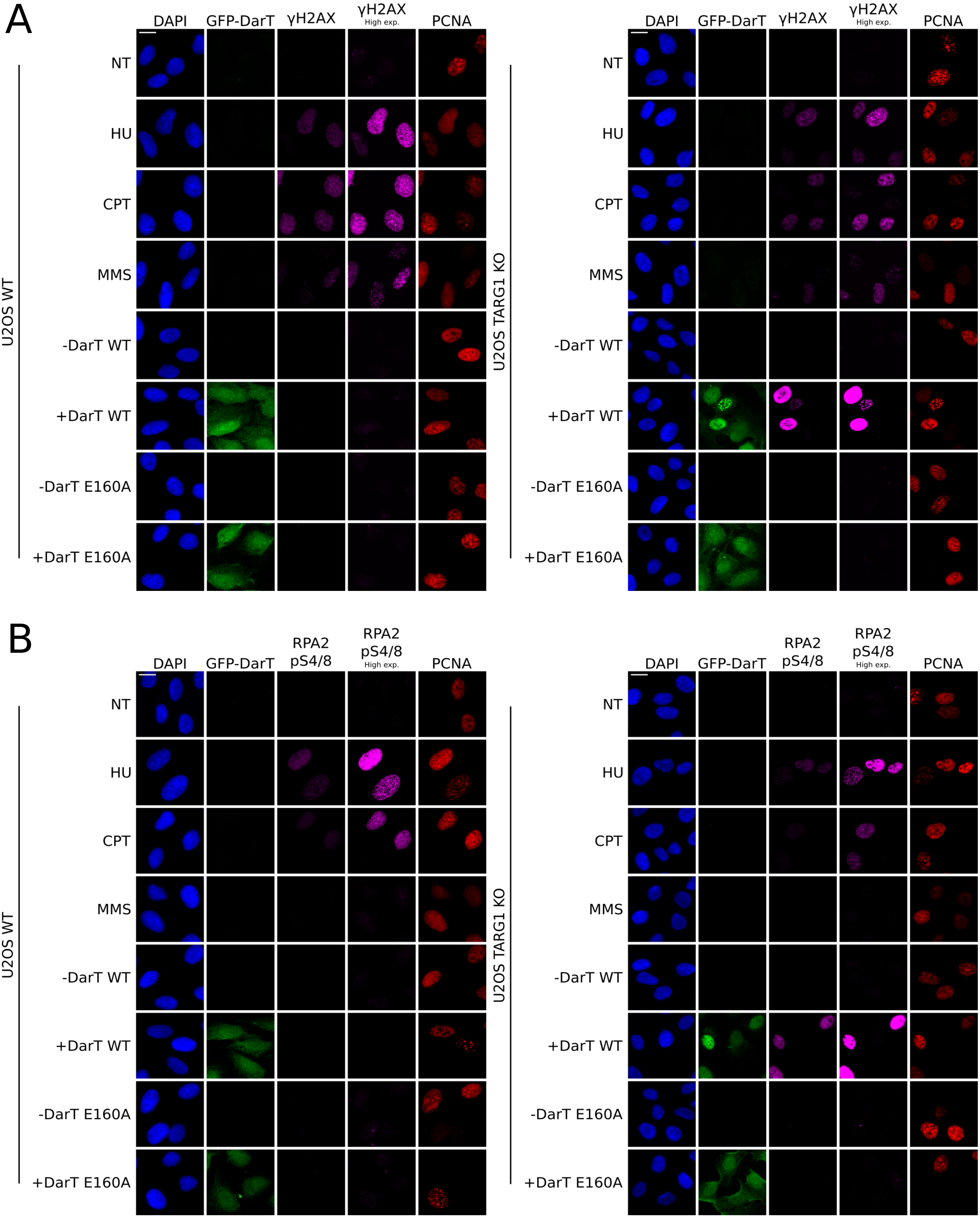
A
DAPI
GFP-DarT
γH2AX
γH2AX High exp.
PCNA
U2OS WT
U2OS TARG1 KO
NT
HU
CPT
MMS
-DarT WT
+DarT WT
-DarT E160A
+DarT E160A
B
RPA2 pS4/8
RPA2 pS4/8 High exp.

Figure S3

Induction of the DDR in DarT-treated cells is found in S-phase and co-localises with PCNA.

A - Extended representative images of both U-2 OS WT and TARG1 KO found in Figure 3A with genotoxin treatments described in Figure 2B.

B - Extended representative images of both U-2 OS WT and TARG1 KO found in Figure 3B with genotoxin treatments described in Figure 2B.

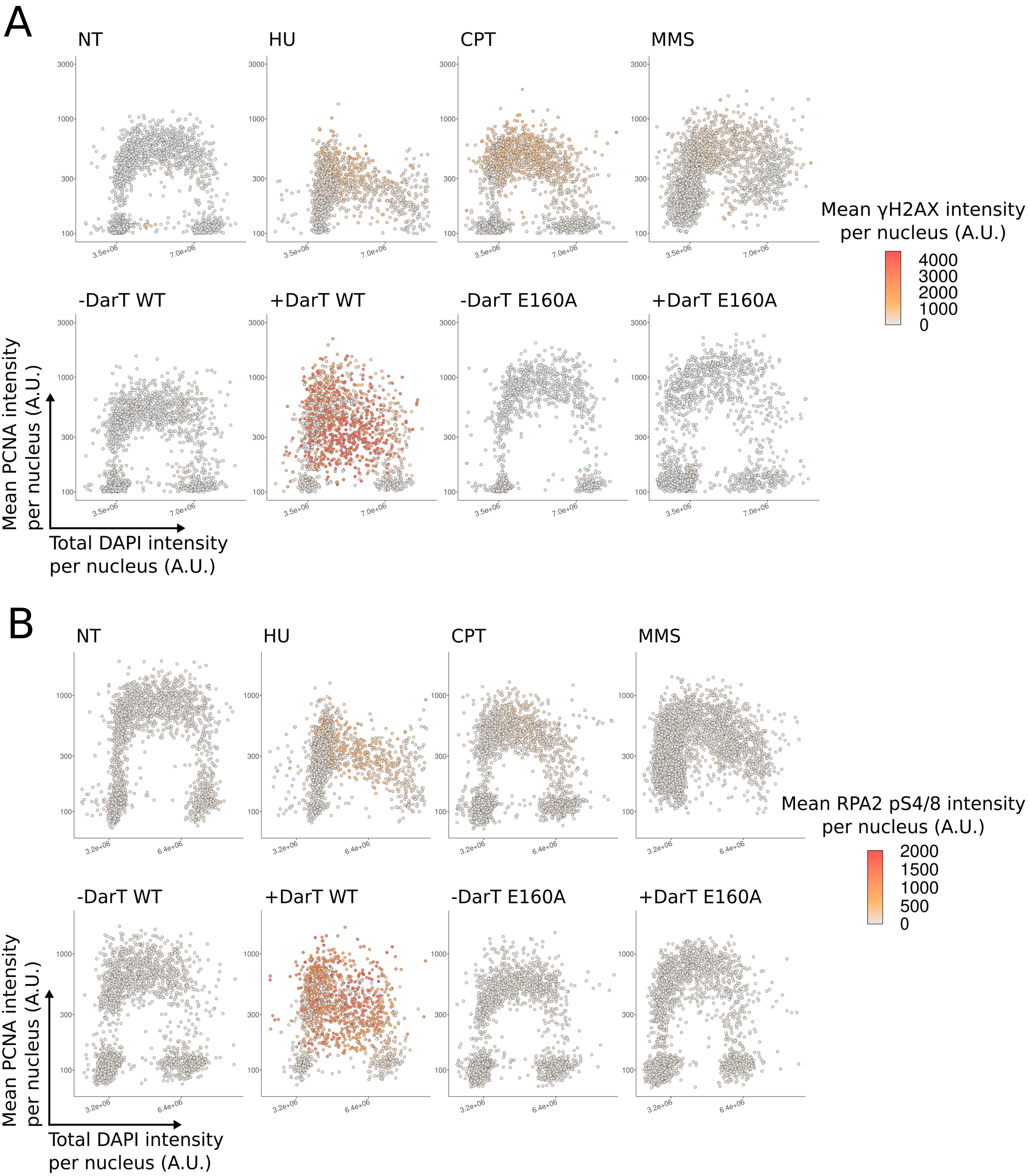

Figure S4

QIBC analysis for TARG1 KO genotoxin treatments.

A – Extended QIBC analysis of Figure 3D for all genotoxin treatments described in Figure 2B in U-2 OS TARG1 KO cells.

B - Extended QIBC analysis of Figure 3F for all genotoxin treatments described in Figure 2B in U-2 OS TARG1 KO cells.

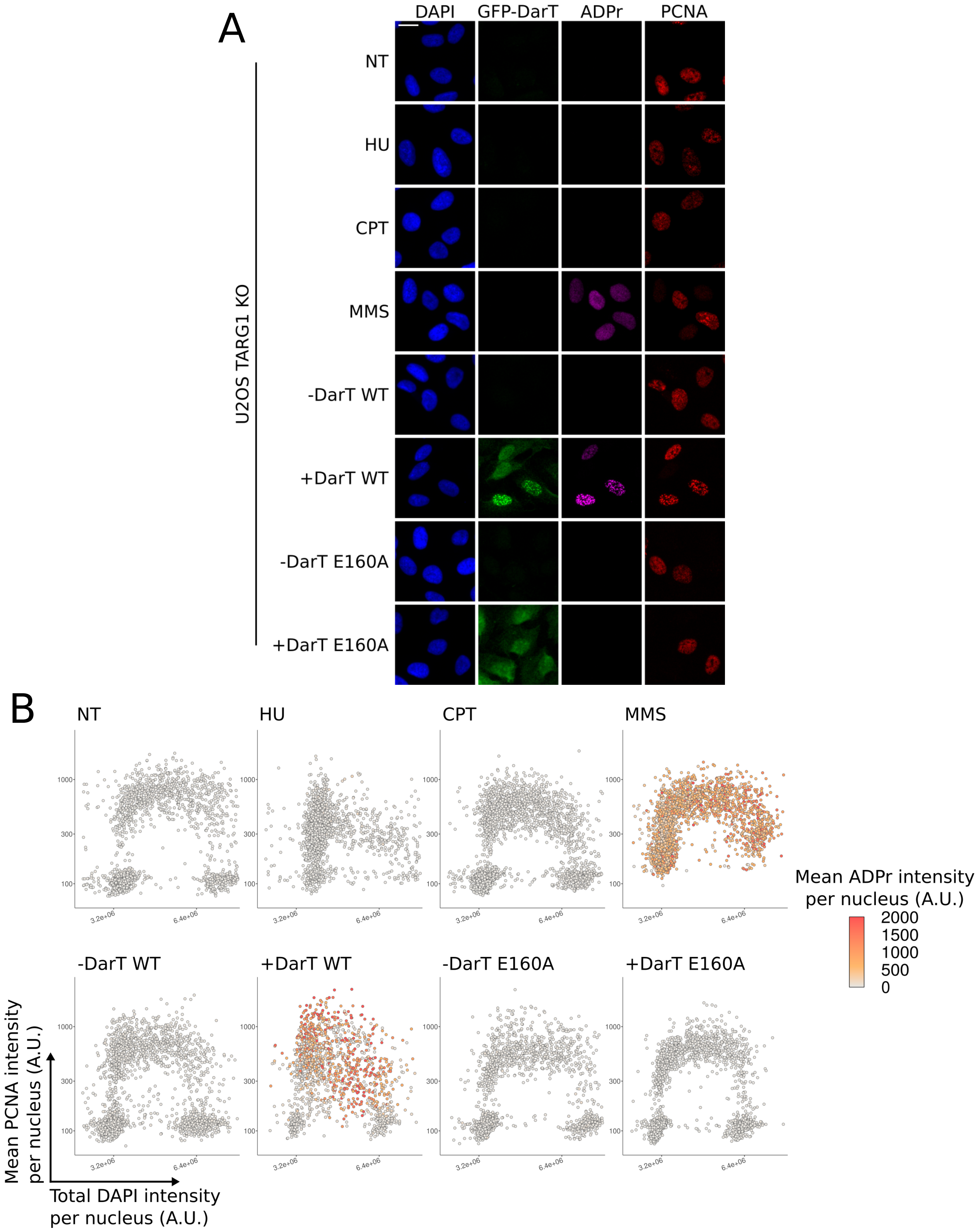

Figure S5

DarT induced ADP-ribosylation is found in replicating cells and co-localises with PCNA.

A - Extended representative images of U-2 OS TARG1 KO found in Figure 4C with genotoxin treatments described in Figure 2B.

B - Extended QIBC analysis of Figure 4E for all genotoxin treatments described in Figure 2B.

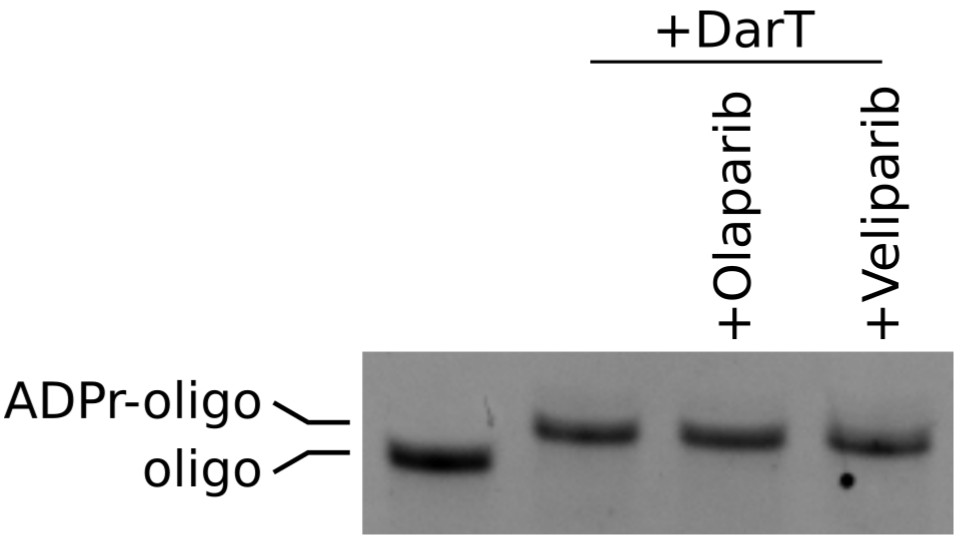

Figure S6
DarT ART activity is not inhibited by olaparib or veliparib in vitro. UV detection of DNA oligonucle-otide ADP-ribosylation by DarT (1 μM) in the presence of olaparib (100 μM) or veliparib (100 μM).

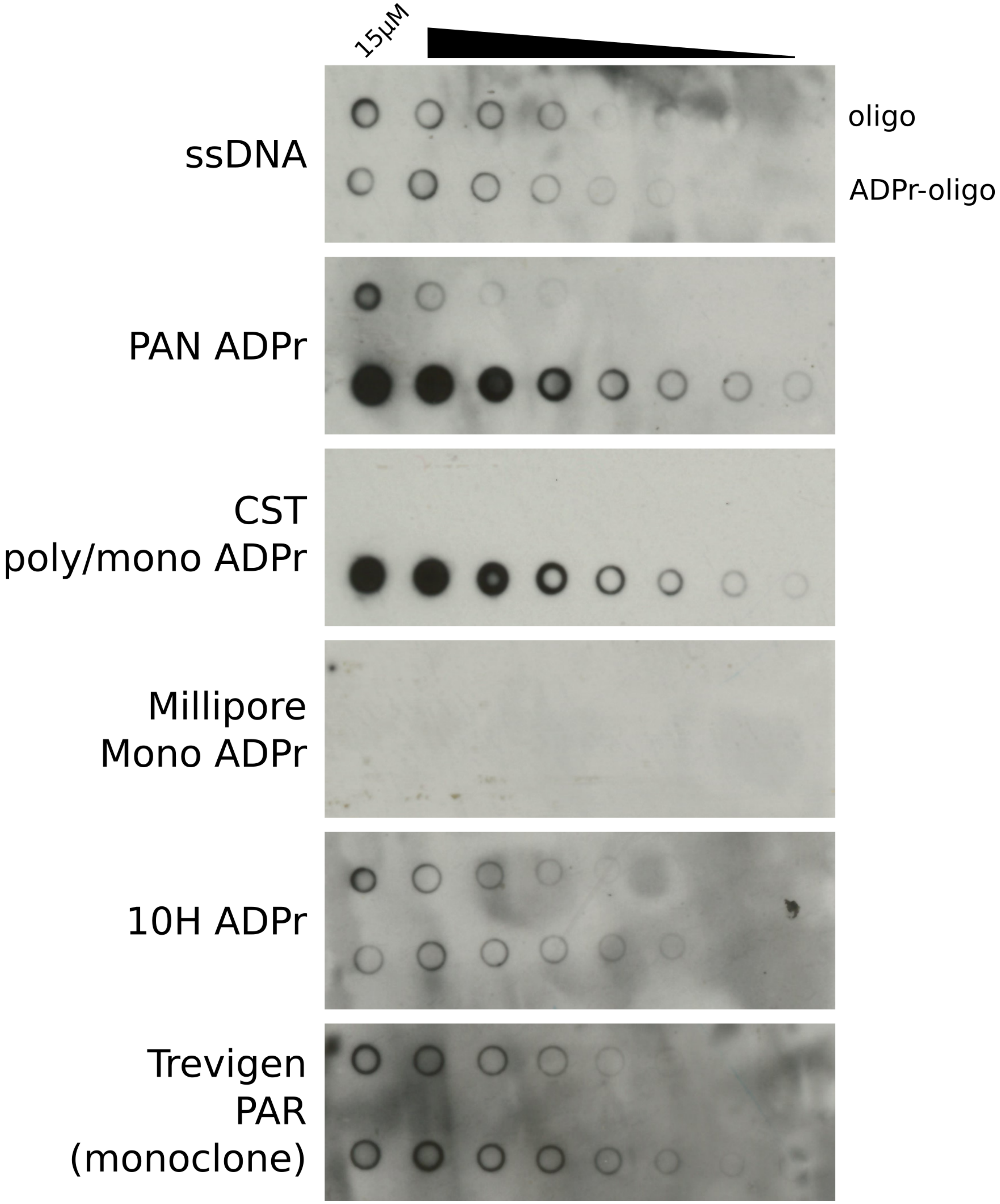

Figure S7
DNA ADP-ribosylation is detectable by CST poly/mono ADP-ribose and PAN-ADP-ribose antibodies. Unmodified or ADP-ribosylation DNA oligonucleotides were dotted onto nitrocellulose membranes at a 2-fold dilution and immunoblotted using the indicated antibodies.
